# Macrophages promote anti-androgen resistance in prostate cancer bone disease

**DOI:** 10.1084/jem.20221007

**Published:** 2023-02-07

**Authors:** Xue-Feng Li, Cigdem Selli, Han-Lin Zhou, Jian Cao, Shuiqing Wu, Ruo-Yu Ma, Ye Lu, Cheng-Bin Zhang, Bijie Xun, Alyson D. Lam, Xiao-Cong Pang, Anu Fernando, Zeda Zhang, Asier Unciti-Broceta, Neil O. Carragher, Prakash Ramachandran, Neil C. Henderson, Ling-Ling Sun, Hai-Yan Hu, Gui-Bo Li, Charles Sawyers, Bin-Zhi Qian

**Affiliations:** 1 Centre for Reproductive Health, College of Medicine and Veterinary Medicine, Queen's Medical Research Institute, The University of Edinburgh, Edinburgh, UK; 2 Human Phenome Institute, Zhangjiang Fudan International Innovation Center, Fudan University, Shanghai, China; 3 BGI-Shenzhen, Shenzhen, China; 4 BGI-Henan, BGI-Shenzhen, Xinxiang, China; 5 Department of Urology, Hunan Cancer Hospital and the Affiliated Cancer Hospital of Xiangya Medicine School, Central South University, Changsha, China; 6 Department of Urology, The Second Xiangya Hospital, Central South University, Changsha, China; 7 Department of Pharmacy, Peking University First Hospital, Beijing, China; 8 Department of Urology, Peking University First Hospital, Beijing, China; 9 Human Oncology and Pathogenesis Program, Memorial Sloan Kettering Cancer Center, New York, NY, USA; 10 Louis V. Gerstner Jr. Graduate School of Biomedical Sciences, Memorial Sloan Kettering Cancer Center, New York City, NY, USA; 11 Edinburgh Cancer Research UK Centre, Institute of Genetics and Cancer, University of Edinburgh, Edinburgh, UK; 12 Centre for Inflammation Research, Queen’s Medical Research Institute, University of Edinburgh, Edinburgh, UK; 13 MRC Human Genetics Unit, Institute of Genetics and Cancer, University of Edinburgh, Edinburgh, UK; 14 Department of Orthopedics, The Second Affiliated Hospital, School of Medicine, Zhejiang University, Hangzhou, China; 15 Shanghai Jiao Tong University Affiliated Sixth People’s Hospital, Shanghai, China; 16 Howard Hughes Medical Institute, Chevy Chase, MD, USA

## Abstract

Metastatic castration-resistant prostate cancer (PC) is the final stage of PC that acquires resistance to androgen deprivation therapies (ADT). Despite progresses in understanding of disease mechanisms, the specific contribution of the metastatic microenvironment to ADT resistance remains largely unknown. The current study identified that the macrophage is the major microenvironmental component of bone-metastatic PC in patients. Using a novel in vivo model, we demonstrated that macrophages were critical for enzalutamide resistance through induction of a wound-healing–like response of ECM–receptor gene expression. Mechanistically, macrophages drove resistance through cytokine activin A that induced fibronectin (FN1)-integrin alpha 5 (ITGA5)–tyrosine kinase Src (SRC) signaling cascade in PC cells. This novel mechanism was strongly supported by bioinformatics analysis of patient transcriptomics datasets. Furthermore, macrophage depletion or SRC inhibition using a novel specific inhibitor significantly inhibited resistant growth. Together, our findings elucidated a novel mechanism of macrophage-induced anti-androgen resistance of metastatic PC and a promising therapeutic approach to treat this deadly disease.

## Teaser

New mechanism of macrophage-driven therapy resistance of metastatic prostate cancer with high ECM expression and SRC activation using a novel in vivo model of bone-metastatic prostate cancer.

## Graphical Abstract

**Figure:**
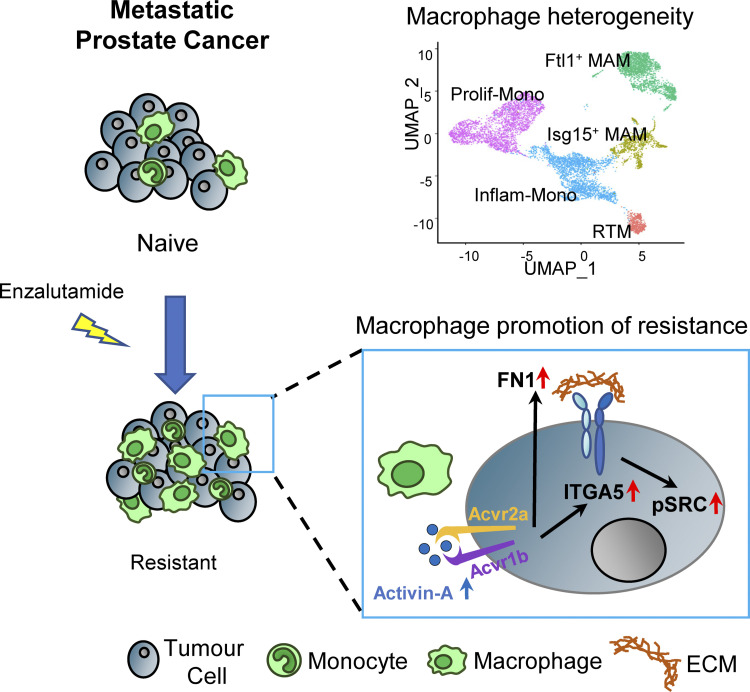

## Introduction

Prostate cancer (PC) is the most common male cancer in the western world (Bray et al., 2018; Rawla, 2019). Metastatic castration-resistant PC (mCRPC) is the final stage of PC that acquires resistance to androgen deprivation therapies (ADT) and accounts for over 90% of PC-related death (Bishr and Saad, 2013; Semenas et al., 2013). Enzalutamide, a second-generation small molecule androgen receptor (AR) antagonist, is one of the modern anti-androgens that significantly improved the survival of patients with mCRPC (Scher et al., 2010). Previous studies have illustrated several tumor cell–intrinsic cell autonomous mechanisms that can lead to anti-androgen resistance, such as *Rb1*;*Trp53* loss–mediated cellular plasticity (Ku et al., 2017; Mu et al., 2017), *CDH1* loss–associated chromatin dysregulation (Zhang et al., 2020), and FAM110B-regulated AR signaling (Vainio et al., 2012).

Tumor microenvironment formed by stromal cells provides extrinsic signals to induce cancer therapy response, which often proceeds accumulation of genetic and epigenetic alterations of tumor cells (Klemm and Joyce, 2015; Quail and Joyce, 2013; Ruffell and Coussens, 2015; Valkenburg et al., 2018). Compared with primary tumor, metastasis-targeted organs have distinct tissue environments in terms of stroma cells, extracellular matrix (ECM), and cytokine milieu (Quail and Joyce, 2013). Previous studies illustrated a clear disparity between primary tumor and metastasis in response to chemotherapy (Fidler et al., 1994; Munoz et al., 2006), which indicated a distinct mechanism involving the metastasis microenvironment in cancer therapy resistance. Indeed, this is supported by recent single-cell transcriptomics studies indicating that gene expression changes rather than the selection of specific resistance clones were observed in patient metastatic PC upon enzalutamide treatment (He et al., 2021). This suggested that the metastasis microenvironment may drive these gene expression changes. However, the vast majority of previous in vivo metastatic PC models (e.g., PC3, C4-2B) are resistant to anti-androgen through tumor cell–intrinsic mechanisms. Therefore, although these models are useful for investigating host factors that promote metastasis, they cannot be used to determine the microenvironmental factors that promote anti-androgen resistance. The mechanistic basis of metastatic microenvironment-induced anti-androgen resistance and the key stromal cell type are largely unknown (Berish et al., 2018; Jinnah et al., 2018; Simmons et al., 2015).

Bone-metastatic PC represents over 70% of all metastatic cases and is highly related to death of patients with advanced PC (Sturge et al., 2011). In the current study, we identified that macrophage is the key stroma cell type significantly enriched in bone-metastatic PC compared with primary tumor and metastases in other organs. Using a newly developed in vivo model of androgen-dependent bone-metastatic PC that can differentiate the processes of anti-androgen resistance from metastasis, our data revealed a novel mechanism of macrophage-induced wound-healing–like response in PC cells with a significant upregulation of ECM and receptor genes. Our study pinpointed the significance of macrophage-derived activin A inducing enzalutamide resistance through the upregulation of a fibronectin (FN1)-integrin alpha 5 (ITGA5) and tyrosine kinase Src (SRC) signaling cascade in PC cells. This is further supported by strong correlations among these key molecular mechanisms in patient transcriptomic datasets, and a significant association with anti-androgen resistance and patient survival. Furthermore, macrophage depletion or SRC inhibition using a novel specific inhibitor significantly impeded the outgrowth of resistant PC in bone. Collectively, our findings elucidated a novel mechanistic link between macrophage-induced wound-healing response and anti-androgen resistance in metastatic disease and suggested novel therapeutic approaches.

## Results

### Specific enrichment of macrophages in bone-metastatic PC is associated with patient survival

**Figure 1.**
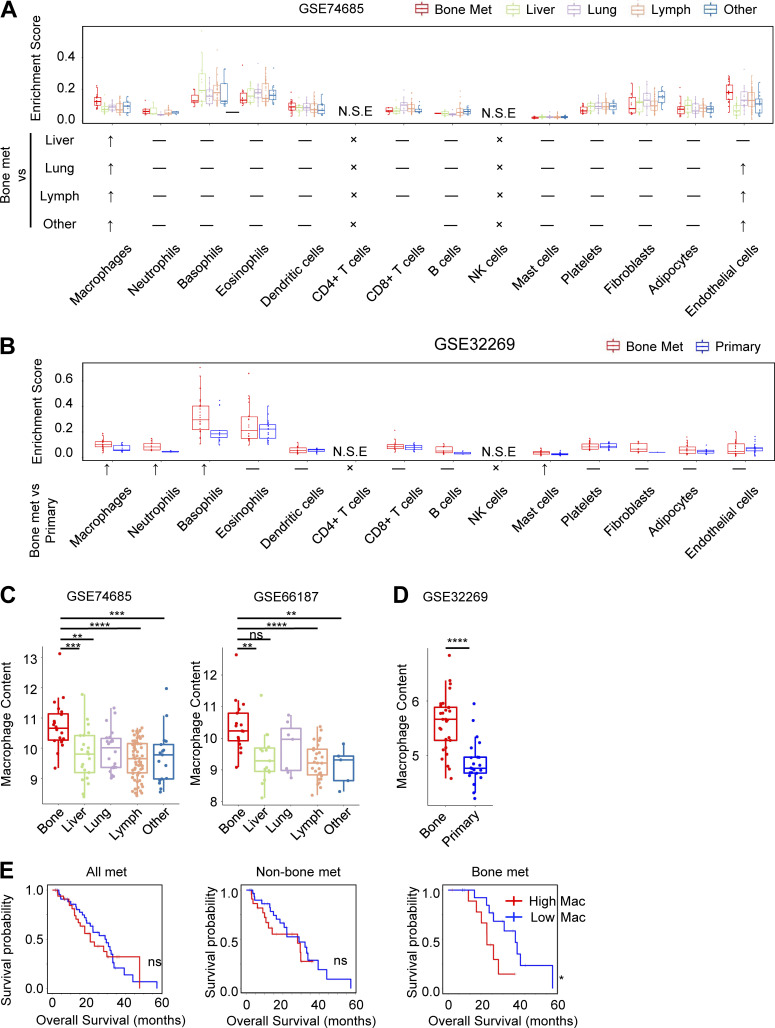
**Specific enrichment of macrophages in bone-metastatic PC is associated with patient survival. (A)** Macrophages are significantly enriched in bone metastasis compared with PC metastasis from other organs. Top: Box plot showing quantification of xCell enrichment score of different stromal cell types in PC metastases in different organs. Bottom: Illustration showing significance of the comparisons: ↑, significantly higher in bone metastasis; —, not significantly different; ×, specific cell type is not present; N.S.E., not significantly estimated with xCell. Significant means P < 0.05 with ANOVA test. **(B)** Macrophages are significantly enriched in bone metastasis compared with PC primary tumor. Top: Box plot showing quantification of xCell enrichment score of different stromal cell types in PC bone metastasis versus primary PC. Bottom: Illustration showing significance of the comparisons: ↑, significantly higher in bone metastasis; —, not significantly different; ×, specific cell type is not present; N.S.E., not significantly estimated with xCell. Significant means P < 0.05 with two-tailed unpaired Student’s *t* test. **(C)** Estimation of macrophage abundance in patient PC bone metastasis and metastases from other organs in indicated datasets. **, P < 0.01; ***, P < 0.001; ****, P < 0.0001; ns, not significant. ANOVA was used. **(D)** Estimation of macrophage abundance in patient PC bone metastasis and primary PC in Gene Expression Omnibus dataset GSE32269. **, P < 0.01; ***, P < 0.001; ****, P < 0.0001; ns, not significant. Two-tailed unpaired Student’s *t* test was used. **(E)** Overall survival of macrophage abundance with median as cut-off in all patients (left), patients without bone metastasis (middle), and patients with bone metastasis (right) in the SU2C dataset. *, P < 0.05; ns, not significant. P value was calculated using the Mantel–Cox test.

To understand the stromal cell components of metastatic PC, we used xCell, a gene signature–based cell type enrichment method, to estimate the relative abundance of various stromal cell populations in previous gene expression datasets that contain primary PC or metastatic PC in different secondary organs (Cai et al., 2013; Haider et al., 2016; Zhang et al., 2015). The enrichment score indicated that macrophage abundance was significantly increased in bone metastasis compared with metastases in other organs (Fig. 1 A) and with primary tumor (Fig. 1 B). Furthermore, using ImSig, an independent immune cell–focused deconvolution algorithm (Nirmal et al., 2018), we confirmed that macrophage abundance was consistently increased in bone metastasis compared with those from other organs (Fig. 1 C) and with primary tumors (Fig. 1 D). Importantly, a recent metastatic PC genomic landscape study with linked longitudinal clinical outcome data (SU2C dataset) provided an opportunity to address whether macrophage abundance was associated with clinical response to next-generation anti-androgen therapy (Abida et al., 2019). Within this landscape study, we identified 56 patients treated with either abiraterone or enzalutamide whose tumor RNA-sequencing (RNA-seq) data were available within 30 d before treatment. Indeed, macrophage abundance estimated by ImSig was significantly associated with poor overall survival in patients with bone-metastatic PC, but not in patients with all different metastasis combined, or in patients with non-bone metastasis (Fig. 1 E).

**Figure S1.**
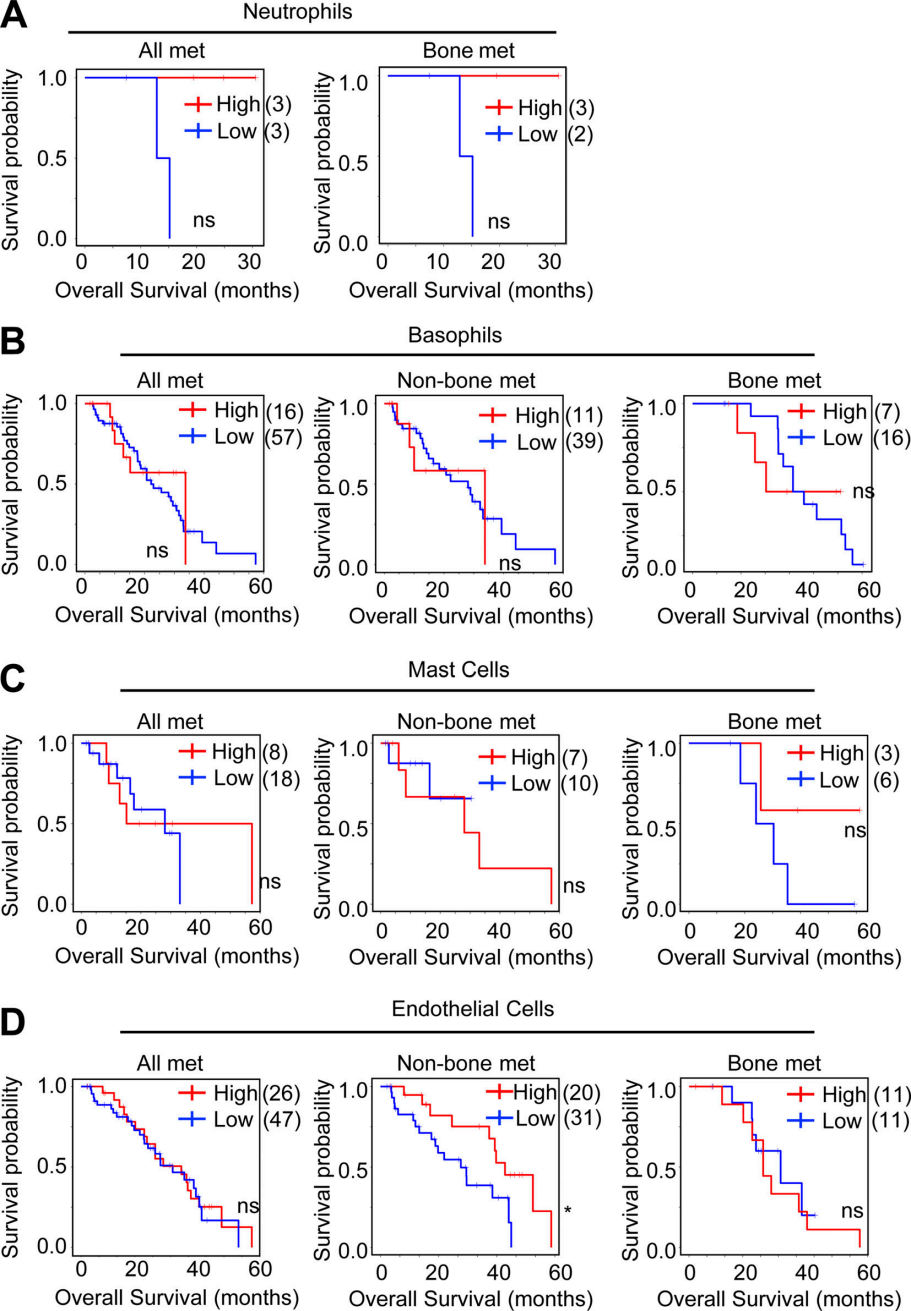
**PC bone metastasis–associated neutrophils, basophils, mast cells, and endothelial cells are not inversely correlated with patient survival. (A)** Kaplan–Meier curve showing association of overall survival with the abundance of neutrophils (estimated using xCell enrichment score) with mean as cut-off in all patients, and patients with bone metastasis in the SU2C dataset. **(B)** Kaplan–Meier curve showing association of overall survival with the abundance of basophils (estimated using xCell enrichment score) with mean as cut-off in all patients, patients with non-bone metastasis, and patients with bone metastasis in the SU2C dataset. **(C)** Kaplan–Meier curve showing association of overall survival with the abundance of mast cells (estimated using xCell enrichment score) with mean as cut-off in all patients, patients with non-bone metastasis, and patients with bone metastasis in the SU2C dataset. **(D)** Kaplan–Meier curve showing association of overall survival with the abundance of endothelial cells (estimated using xCell enrichment score) with mean as cut-off in all patients, patients with non-bone metastasis, and patients with bone metastasis in the SU2C dataset. *, P < 0.05; ns, not significant. P value was calculated using Mantel–Cox test.

In addition to macrophages, we also observed that endothelial cells were enriched in bone metastasis compared with metastasis in other organs (expect for liver metastasis; Fig. 1 A) and that neutrophils, basophils, and mast cells were enriched in bone metastasis compared with primary tumor (Fig. 1 B). We further analyzed the correlation of the abundance of these cells with patient survival using the SU2C dataset. Neutrophils can be detected in only six samples using the xCell algorithm, suggesting the low infiltration of neutrophils in metastatic PC samples. Among the six samples, five of them were bone metastasis and the other one was soft tissue metastasis. Thus, we can only analyze the correlation of neutrophil abundance with the survival of all patients and patients with bone metastasis, but not soft tissue metastasis. High abundance of neutrophils and endothelial cells was significantly correlated with longer survival of patients with metastatic PC in all samples (Fig. S1 A, left) and patients with non-bone metastasis (Fig. S1 D, middle), respectively. No significant correlation was observed between the abundance of neutrophil, basophil, mast cell, and endothelial cells and patient survival in all other analyses (Fig. S1, A–D). Collectively, these data suggested a specific role of macrophages in bone-metastatic PC.

### Enzalutamide resistance of bone-metastatic PC is dependent on macrophages

**Figure 2.**
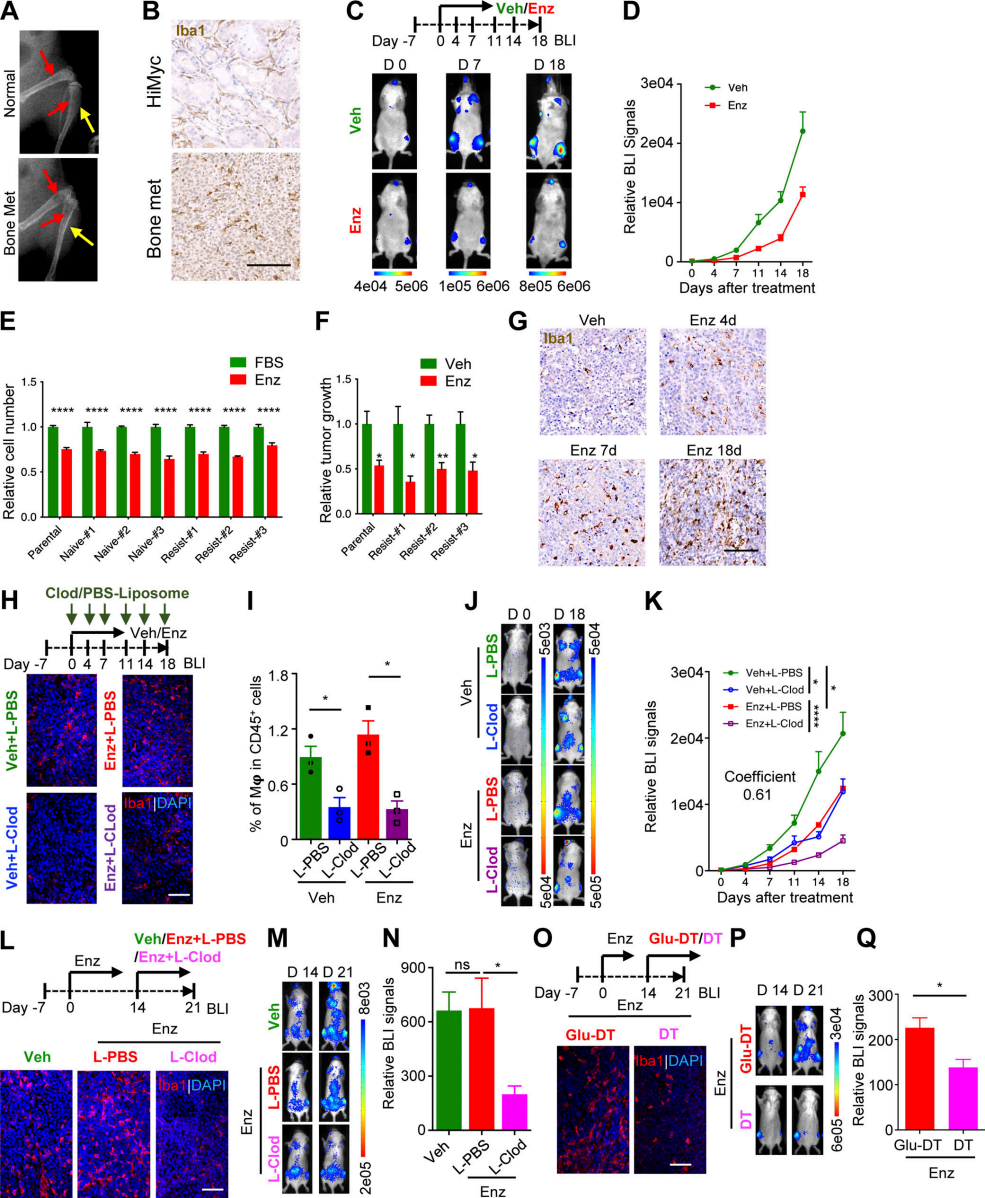
**Enzalutamide resistance of bone-metastatic PC is dependent on macrophages. (A)** Representative x-ray images showing mixed osteolytic and osteoblastic MycCaP-Bo bone metastasis lesion compared with normal bone. Bone marrow region and bone matrix are indicated by red and yellow arrows, respectively. **(B)** Representative immunohistochemistry staining of mouse macrophage marker Iba1 in samples of HiMyc primary prostate tumor (HiMyc) and MycCaP-Bo–derived bone metastasis (Bone met). **(C)** Representative BLI of bone metastasis receiving daily treatment of vehicle (Veh) or enzalutamide (Enz) on days 0, 7, and 18 following the treatment schematic diagram shown on top. **(D)** Quantification of BLI signals of bone metastasis of hind legs (see Materials and methods) in mice with indicated treatment (*n* = 12∼14). **(E)** In vitro response to enzalutamide of MycCaP-Bo cells recovered from in vivo enzalutamide naive (Naive #1–3) or resistant (Resist #1–3) bone metastasis compared with parental MycCaP-Bo cells as measured by relative growth (*n* = 3). **(F)** In vivo response to enzalutamide of bone metastasis derived from MycCaP-Bo cells recovered from enzalutamide-resistant bone metastasis (Resist #1–3) compared with bone metastasis of parental MycCaP-Bo cells on day 18 as measured by relative BLI signal (*n* = 6∼10). **(G)** Representative images of Iba1 staining in MycCaP-Bo bone metastasis with indicated treatment. **(H)** Macrophage depletion by L-Clod inhibits the development of enzalutamide resistance of PC bone metastasis. Representative images of Iba1 whole-mount staining of bone metastasis samples collected on day 18 with indicated treatment. **(I)** FACS quantification of percentage of F4/80^+^ macrophages in total CD45^+^ cells of bone metastasis samples collected on day 18 with indicated treatment. **(J)** Representative BLI of mice from H on day 0 and day 18. **(K)** Quantification of BLI signals of bone metastasis in mice receiving treatments as indicated in H (*n* ≥ 10). Coefficient of drug interaction on day 18 equals 0.61, indicating significant synergistic effect. **(L)** Representative images of whole-mount staining of Iba1 in bone metastasis samples collected on day 21 with indicated treatment as shown in the diagram on top. **(M)** Representative BLI of mice from L on day 14 and day 21. **(N)** Quantification of BLI signal of bone metastasis from L on day 21 relative to day 14 receiving indicated treatments (*n* = 8∼10). Shown as relative signal of bone metastasis at day 21 normalized to same tumor at day 14. **(O)** Representative images of whole-mount staining of Iba1 in bone metastasis samples collected on day 21 in CD11b-DTR bone marrow mosaic mice with indicated treatment, as shown in the diagram on top. **(P)** Representative BLI of mice from O on day 14 and day 21. **(Q)** Quantification of BLI signal of bone metastasis on day 21 relative to day 14 in mice from O (*n* = 6). Data are mean ± SEM; *, P < 0.05; **, P < 0.01; ***, P < 0.001; ****, P < 0.0001; ns, not significant. ANOVA was used in N, and two-tailed unpaired Student’s *t* test was used in the rest of the analyses. Scale bar = 100 μm.

**Figure S2.**
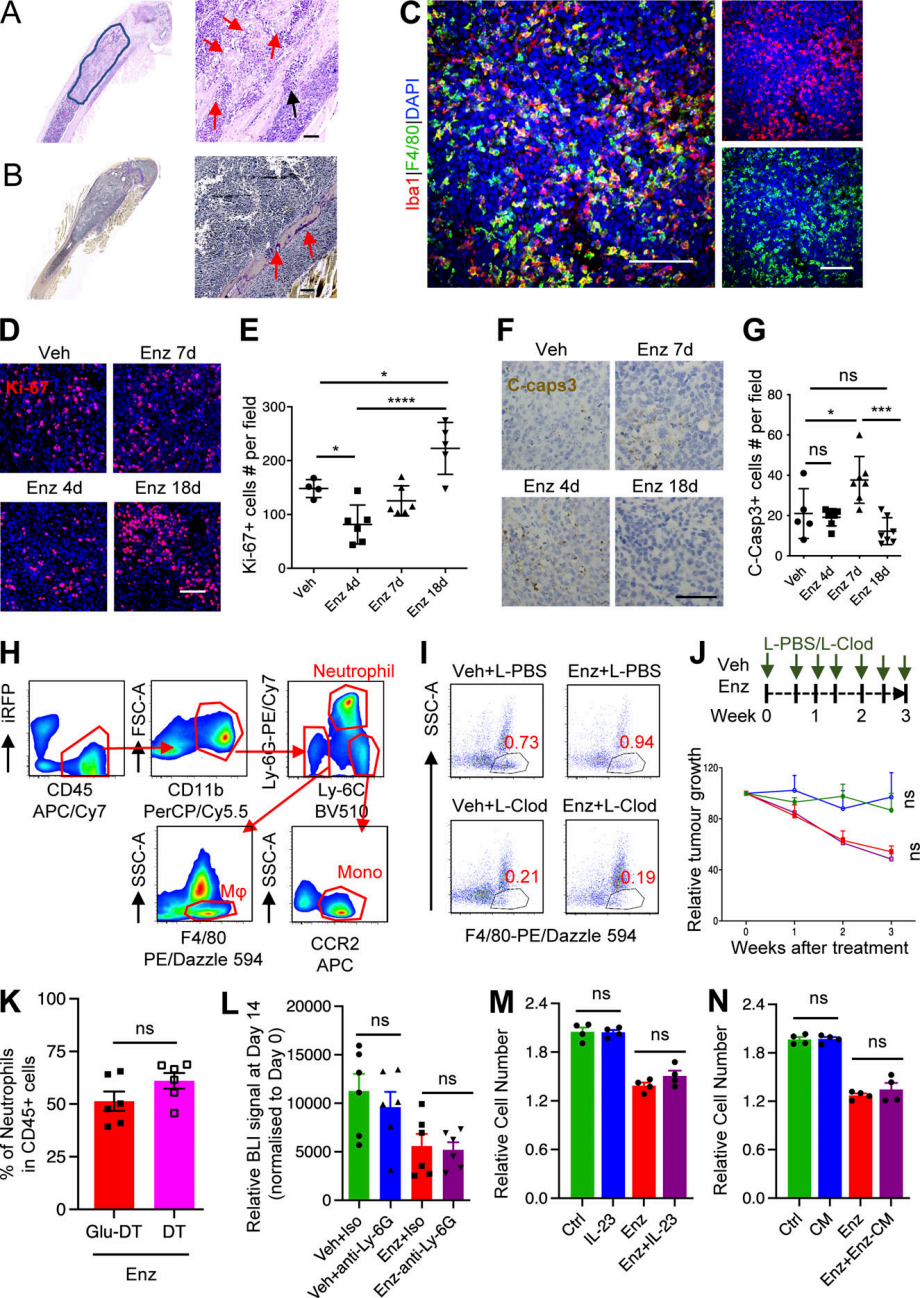
**Neutrophils contribute minimally to anti-androgen resistance. **Data related to Fig. 2. **(A)** Representative H&E staining of MycCaP-Bo bone metastasis sample. Circled area indicates the tumor region. Red arrows indicate newly formed bone matrix; black arrow indicates bone absorption area. **(B)** Representative TRAP staining of MycCaP-Bo bone metastasis. Red arrows indicate TRAP^+^ osteoclasts. **(C)** Representative image of Iba1 (red) and F4/80 (green) IF staining in bone metastasis lesion showing the specificity of Iba1 as the macrophage marker. **(D)** Representative images of Ki-67 staining of bone metastasis lesions with treatment of vehicle (Veh) or enzalutamide (Enz) at indicated time points. **(E)** Quantification of Ki-67 staining in bone metastasis lesions with treatment of vehicle or enzalutamide at indicated time points. **(F)** Representative images of cleaved caspase-3 staining of the same samples as in D and E. **(G)** Quantification of cleaved caspase-3 staining in the same samples as in D and E. **(H)** Gating strategy for identification of F4/80^+^ macrophages, CCR2^+^ Inflam-Monos, and neutrophils in bone metastasis. **(I)** Representative flow cytometry dot plots showing macrophage depletion using liposomal clodronate, shown as the percentage of F4/80^+^ cells (gated cells) in CD45^+^ total cells. **(J)** Relative growth of spontaneous tumor in HiMyc mice under vehicle or enzalutamide combined with the treatment of liposome PBS (L-PBS) or L-Clod following the schematic diagram on top. **(K)** Quantification showing the percentage of neutrophils (gated as CD45^+^CD11b^+^Ly-6G^+^, shown as in Fig. S2 H) in total CD45^+^ cells from bone metastasis samples collected on day 21 with DT and Glu-DT (control toxin) treatment (*n* = 6). **(L)** Quantification of relative tumor growth on day 14 after treatments (normalized to day 0) showing that neutrophil depletion using anti-Ly-6G Ab did not affect anti-androgen response in vivo. MycCaP-Bo bone metastasis received daily treatment of vehicle or enzalutamide plus isotype (Iso) or neutrophil-depleting Abs (Anti-Ly-6G, 200 mg/mouse, i.p. injection, twice a week; *n* = 6). **(M)** Relative cell number of MycCaP-Bo cells upon 4 d of indicated treatments revealed that IL-23 did not affect enzalutamide response in vitro. Enzalutamide pre-treated MycCaP-Bo cells were further treated with normal medium (Ctrl), IL-23 alone (100 ng/ml), enzalutamide alone (Enz, 1 mM), and enzalutamide plus IL-23 (Enz+IL-23), followed by MTT assay on day 4 of treatments (*n* = 4). **(N)** Relative cell number of MycCaP-Bo cells upon 4 d of indicated treatments revealed that MDSC-conditioned medium did not affect enzalutamide response in vitro. Enzalutamide–pre-treated MycCaP-Bo cells were further treated with normal culture (Ctrl), MDSC-conditioned medium alone (CM), enzalutamide alone (Enz, 1 mM), and enzalutamide plus enzalutamide-primed MDSC-conditioned medium (Enz+Enz-CM), followed by MTT assay on day 4 of treatments (*n* = 4). Data are mean ± SEM; *, P < 0.05; ***, P < 0.001; ****, P < 0.0001; ns, not significant. ANOVA was used in E and G, and two-tailed unpaired Student’s *t* test was used in J–N. Scale bar = 100 μm.

To investigate the role of bone metastasis microenvironment in anti-androgen resistance, we developed a new model, MycCaP-Bo, through three rounds of in vivo selection of bone homing cells following intra-cardiac inoculation of an androgen-dependent murine PC cell line, MycCaP. The MycCaP cells were originally derived from Myc oncogene–driven HiMyc tumor model in FVB-syngeneic background (Watson et al., 2005). MycCaP-Bo cells were labeled with a lentiviral vector that expresses firefly luciferase and an infra-red fluorescent protein (iRFP; Filonov et al., 2011) to allow in vivo detection with bioluminescent imaging (BLI) and ex vivo analysis with flow cytometry, respectively. BLI images indicated that MycCaP-Bo cells formed specific bone lesions commonly in calvaria, jaw, spine, limb bones, but most frequently in hind legs. These lesions resemble a mixed osteogenic and osteolytic pathology resembled that of patient diseases as illustrated by x-ray scanning (Fig. 2 A), H&E histology staining (Fig. S2 A), and tartrate-resistant acid phosphatase (TRAP) staining for osteoclasts (Fig. S2 B). Consistent with the observation in patients (Fig. 1, B and D), tumor-infiltrating macrophages were significantly higher in MycCaP-Bo bone metastasis compared with the primary tumor of the original HiMyc model, as measured by staining of macrophage marker Iba1 (Fig. 2 B). Iba1 has been widely used to stain most macrophage populations (except alveolar macrophages; Linde et al., 2018; Kohler, 2007; DeFalco et al., 2015) and colocalized with classical macrophage marker F4/80 in our model (Fig. S2 C). For simplicity, all BLI quantifications in the current study were focused on hind legs (detailed in the Materials and methods). BLI quantification indicated that MycCaP-Bo bone lesions responded to enzalutamide initially (before day 14) and progressed to resistance after 14 d of enzalutamide treatment, indicated by the comparable growth rate quantified by the fold increase of BLI signal intensity from day 14 to day 18 (vehicle [Veh]: 2.13; enzalutamide [Enz]: 2.87; Fig. 2, C and D). Consistently, at cellular level, enzalutamide significantly inhibited tumor cell proliferation as measured by Ki-67 staining after 4 d into the treatment (Fig. S2, D and E) and induced apoptosis as measured by cleaved caspase 3 staining after 7 d into the treatment (Fig. S2, F and G) in the MycCaP-Bo model. In contrast, enzalutamide-resistant tumors (Enz 18 d) showed increased proliferation and comparable apoptosis rates compared with naive tumors (Fig. S2, D–G).

To understand the mechanism of resistance, we started by comparing MycCaP-Bo cells isolated from resistant tumors with those from naive tumors. To our surprise, three different batches of tumor cells harvested from three independent resistant tumors responded to enzalutamide treatment in vitro to the same level as cells from naive tumors and the parental cells (Fig. 2 E). When re-inoculated in vivo, bone metastasis formed by MycCaP-Bo cells recovered from previously resistant tumors also responded to enzalutamide to a similar extent compared with tumors generated by the parental MycCaP-Bo cells (Fig. 2 F). Together, these data suggested that the resistance in vivo is unlikely to be caused by genetic alteration in tumor cells, but rather caused by a tumor cell non-autonomous mechanism that develops over time through interaction with the in vivo metastasis microenvironment.

Given the role of macrophages in cancer therapy resistance (Klemm and Joyce, 2015; Ruffell and Coussens, 2015; Coffelt and de Visser, 2015) and their specific involvement in MycCaP-Bo bone metastasis from our analysis (Fig. 1), we stained macrophages using Iba1 and found enhanced infiltration of macrophages in MycCaP-Bo bone metastasis upon enzalutamide treatment (Fig. 2 G). Consequently, we decided to test the role of macrophages in our MycCaP-Bo model using the classical chemical method of macrophage depletion with clodronate liposome (L-Clod; Gordon and Taylor, 2005; Fig. 2, H and I; and Fig. S2, H and I). This macrophage depletion significantly enhanced the effect of enzalutamide on established MycCaP-Bo bone metastasis in a highly synergistic manner (coefficient of drug interaction = 0.61; Fig. 2, J and K). In contrast, macrophage depletion using L-Clod did not enhance the efficacy of enzalutamide in primary HiMyc tumors (Fig. S2 J). These data confirmed that metastasis-associated macrophages (MAMs) are particularly important for the development of enzalutamide resistance of MycCaP-Bo–derived bone-metastatic PC. To further determine whether MAMs are critical for the continuous growth of resistant tumors, we generated resistant tumors by treating mice with enzalutamide for 14 d, then split them into three groups treated with vehicle, enzalutamide only, or enzalutamide plus L-Clod, respectively (Fig. 2 L). In this setting, the tumor growth was not different between vehicle and enzalutamide treatment (Fig. 2, M and N), confirming that these bone lesions indeed became resistant. Macrophage ablation significantly inhibited the growth of resistant tumors (Fig. 2, M and N), indicating that this resistance is dependent on the continuous presence of MAMs. To further confirm our findings with L-Clod, we used a genetic model of macrophage ablation with a syngeneic FVB transgenic mouse expressing the human diphtheria toxin (DT) receptor (DTR, also known as heparin-binding EGF or hb-EGF) under the control of a truncated mouse CD11b promoter (CD11b-DTR). In these mice, CD11b^+^F4/80^+^ macrophages, but not neutrophils (also CD11b expressing), can be conditionally ablated upon DT injection (Duffield et al., 2005). As established previously (Qian et al., 2009), mosaic mice were generated by bone marrow transplantation using CD11b-DTR mice as the bone marrow donors in order to avoid potential leaky expression of the transgene in non-hematopoietic cells. Resistant MycCaP-Bo bone metastases were established in these mosaic mice through 14 d of enzalutamide treatment, then divided into two groups to receive enzalutamide plus DT or mutated DT (Glu^52^-DT) as toxin control (Hu et al., 1998). DT treatment led to efficient depletion of macrophages in these tumors (Fig. 2 O) significantly inhibited resistant tumor growth (Fig. 2, P and Q), without leaky depletion of neutrophils as expected (Fig. S2 K). Collectively, data from these two independent models indicated that MAMs are critical for enzalutamide resistance of the MycCaP-Bo model of bone-metastatic PC.

Previous study demonstrated that IL-23 derived from polymorphonuclear myeloid-derived suppressor cells (MDSCs; also known as neutrophils) could promote castration resistance in models of primary PC (Calcinotto et al., 2018). We further examined whether such mechanism is involved in the anti-androgen resistance in our MycCaP-Bo model. To this end, we first used anti-Ly-6G antibodies (Abs) to deplete neutrophils in bone metastasis in vivo and monitored the tumor growth. Our results showed no difference in bone metastasis outgrowth and enzalutamide resistance between neutrophil depletion and control groups (Fig. S2 L). Furthermore, neither recombinant IL-23 nor murine bone marrow MDSC–conditioned medium was able to promote enzalutamide resistance of MycCaP-Bo cells in vitro, following the protocol of the previous study (Fig. S2, M and N). Consistently, the expression level of *Il23ra* (the murine receptor for IL-23) was non-detectable in bulk RNA-seq data of Myc-CaP-Bo cells from in vitro culture, or from in vivo bone metastases at naive or resistant stage (data not shown). Together, these in vivo and in vitro data demonstrated that neutrophils and IL-23 are dispensable for the anti-androgen resistance of the MycCaP-Bo bone metastasis.

### Enzalutamide resistance depends on both monocyte-derived and bone-resident macrophages

**Figure 3.**
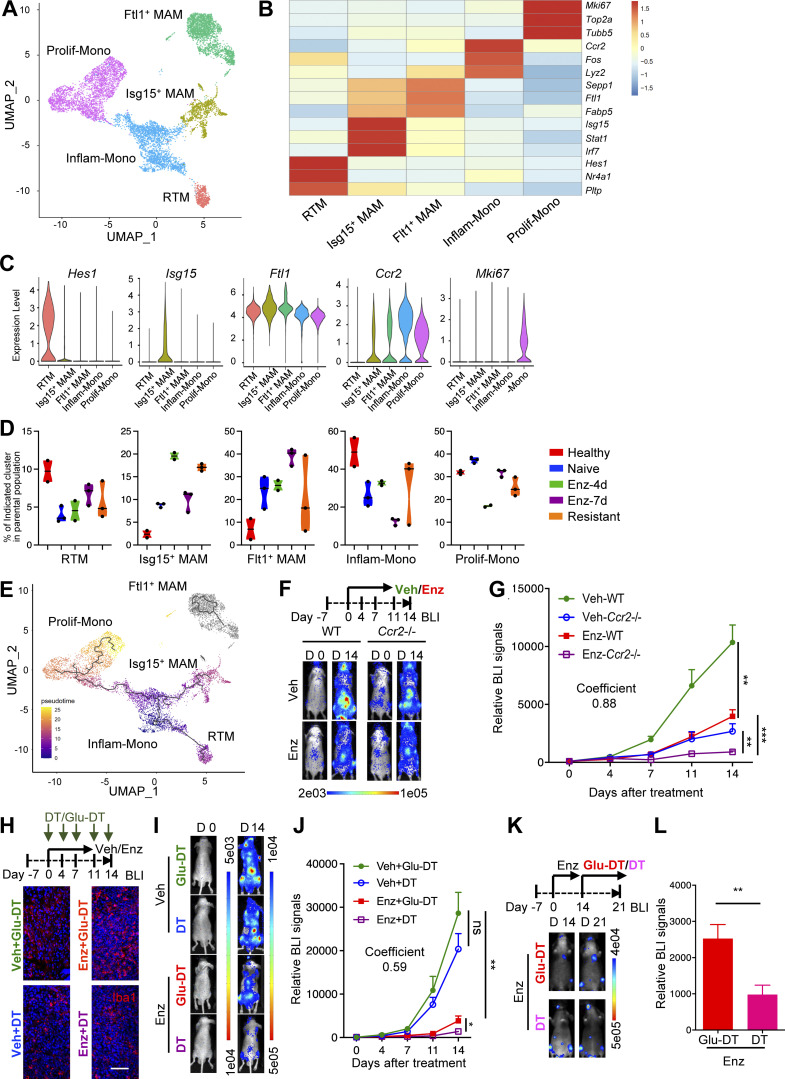
**Enzalutamide resistance depends on both monocyte-derived and bone-resident macrophages. (A)** UMAP of monocyte/macrophage populations from all samples. All cells are colored by their cell types. **(B)** Heatmap showing the expression of representative genes for each population. **(C)** Expression level of featured genes in each population. **(D)** Box plots showing the percentage of each population in total monocyte/macrophage across different treatment groups. Healthy (*n* = 2), naive tumor (*n* = 3), enzalutamide 4 d (Enz-4d, *n* = 2), enzalutamide 7 d (Enz-7d, *n* = 3), and enzalutamide 18 d (Resistant, *n* = 3). **(E)** Pseudotime analysis of all monocyte/macrophage populations by Monocle3. All cells were colored by pseudotime score. **(F)** Deficiency of monocyte derived macrophage in *Ccr2*^*−/−*^ mice inhibits enzalutamide resistance of MycCaP-Bo bone metastasis. Representative BLI of MycCaP-Bo bone metastasis receiving daily treatment of vehicle (Veh) or enzalutamide (Enz) in WT and CCR2-knockout (*Ccr2*^−/−^) mice (*n* = 6∼14). **(G)** Quantification of MycCaP-Bo bone metastasis as indicated in F (*n* = 6∼14). Coefficient of drug interaction = 0.88 on day 14 indicating significant synergistic effect. **(H)** Depletion of bone marrow–resident macrophage in CD169-DTR mice delayed enzalutamide resistance of MycCaP-Bo bone metastasis. Representative images of Iba1 staining in bone metastasis samples collected on day 14 with indicated treatment as shown in the diagram on top. Glu-DT, control mutant toxin. **(I)** Representative BLI of bone metastasis in mice at specified time points receiving indicated treatments. Glu-DT, control mutant toxin. **(J)** Quantification of bone metastasis in mice at specified time points receiving indicated treatments (*n* = 6∼16). Coefficient of drug interaction on day 14 indicating significant synergistic effect. Glu-DT, control mutant toxin. **(K)** Macrophage depletion in CD169-DTR mice blocked growth of resistant bone metastases. Representative BLI of bone metastasis on day 14 and day 21 in mice receiving indicated treatments. **(L)** Quantification of BLI of bone metastasis on day 21 relative to day 14 in mice receiving indicated treatments as indicated in K (*n* = 6). Data in D are median ± quartiles; all other data are mean ± SEM. *, P < 0.05; **, P < 0.01; ***, P < 0.001; ns, not significant. Calculated by two-tailed unpaired Student’s *t* test. Scale bar = 100 μm.

**Figure S3.**
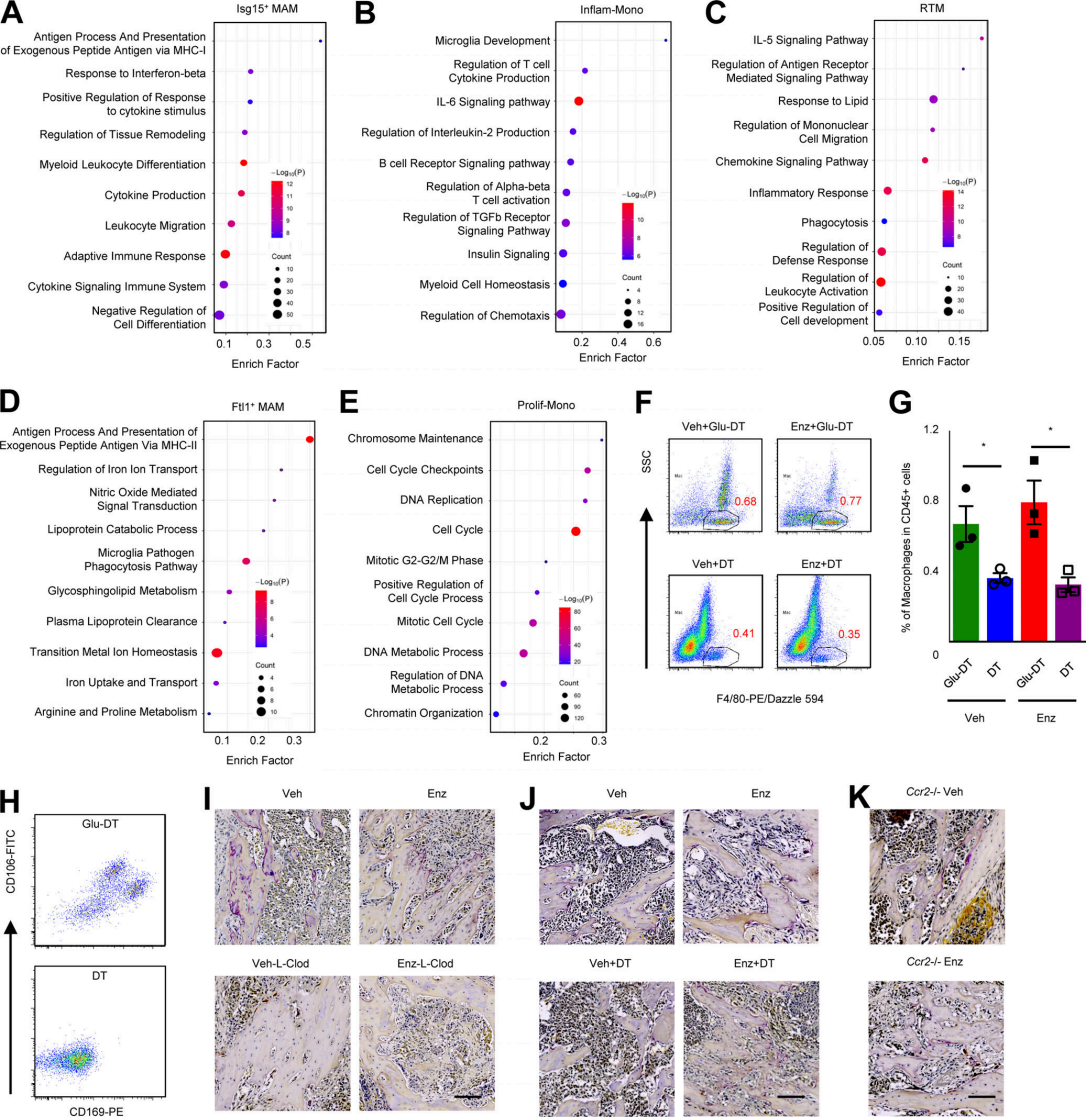
**Macrophage sub-populations in bone-metastatic PC. **Data related to Fig. 3. **(A)** Enriched pathways of *Isg15*^+^ MAM based on differentially expressed genes. **(B)** Enriched pathways of Inflam-Mono based on differentially expressed genes. **(C)** Enriched pathways of RTM based on differentially expressed genes. **(D)** Enriched pathways of *Ftl1*^+^ based on differentially expressed genes. **(E)** Enriched pathways of proliferating monocyte (Prolif-Mono) based on differentially expressed genes. **(F)** Representative flow cytometry dot plots showing the percentage of SSC^lo^F4/80^+^ macrophages (gated cells) in total CD45^+^ cells from bone metastasis samples in CD169-DTR mice collected on day 14 with DT and Glu-DT (control toxin) treatment (*n* = 3). **(G)** Quantification of FACS data showing the percentage of SSC^lo^F4/80^+^ macrophages (gated as in F) in total CD45^+^ cells from bone metastasis samples in CD169-DTR mice collected on day 14 with DT and Glu-DT (control toxin) treatment (*n* = 3). **(H)** Representative flow cytometry dot plots showing the depletion efficiency of bone marrow resident macrophages (CD169^+^CD106^+^) of all F4/80^+^ macrophages from F. **(I)** Representative TRAP staining of MycCaP-Bo bone metastasis in WT mice with indicated treatments for 18 d. **(J)** Representative TRAP staining of MycCaP-Bo bone metastasis in CD169-DTR mice with indicated treatments for 18 d. **(K)** Representative TRAP staining of MycCaP-Bo bone metastasis in *Ccr2*^−/−^ mice with indicated treatments for 18 d. Data are mean ± SEM in G; *, P < 0.05. P value was calculated using two-tailed unpaired Student’s *t* test. Scale bar = 100 μm.

Macrophages derived from embryonic precursor cells (resident-tissue macrophages) and bone marrow monocytes (monocyte-derived macrophages [MDMs]) may exhibit distinct functions in cancer (Schulz et al., 2012; Jacome-Galarza et al., 2019). For example, our recent studies showed that macrophages derived from Ly-6C^+^ inflammatory monocytes (Inflam-Monos), but not CD169^+^ bone-resident macrophages, are important for breast cancer bone metastasis growth (Ma et al., 2020). To characterize macrophage heterogeneity in our MycCaP-Bo model, we performed single-cell RNA-seq (scRNA-seq) of cells from healthy bone marrow, bone metastasis with vehicle treatment (naive), enzalutamide for 4 d (Enz-4d), 7 d (Enz-7d), and 18 d (resistant). Through Uniform Manifold Approximation and Projection (UMAP) clustering, we identified a total of 9,454 monocytes/macrophages that were further divided into five subsets based on the differentially expressed genes (Fig. 3 A). According to their signature gene expression, we defined these subsets as proliferating monocytes (*Mki67*), Inflam-Monos (*Ccr2*, *Fos*), resident tissue macrophages (RTMs; *Hes1*, *Nr4a1*), Isg15^+^ macrophages (*Isg15*, *Stat1*, *Irf7*), and Ftl1^+^ macrophages (*Ftl1*, *Fabp5*; Fig. 3, B and C). Among different samples, the abundance of RTMs and Inflam-Monos were higher in normal bone marrow (healthy) compared with bone metastasis (naive; Fig. 3 D), which agreed with the identification as RTM and Inflam-Mono from gene signature. In contrast, the other two macrophage populations increased in bone metastasis samples compared with normal, suggesting that they are MAMs (Fig. 3 D). Upon enzalutamide treatment, Isg15^+^ MAMs further increased in resistant tumors compared with naive tumors, while Ftl1^+^ MAMs abundance was not significantly different, despite some fluctuation at day 7 and large variation among samples at day 18 (Fig. 3 D). Pseudotime analysis indicated that Isg15^+^ MAMs were MDMs and potentially differentiated from Inflam-Monos (Fig. 3 E). Further pathway enrichment analysis identified major pathways enriched in different macrophage subsets (Fig. S3, A–E). Notably, Isg15^+^ MAMs and Inflam-Monos were predominantly enriched for inflammatory pathways, including positive regulation of response to cytokine, regulation of tissue remodeling, and cytokine production for Isg15^+^ MAMs (Fig. S3 A), and regulation of T cell cytokine production and regulation of α-β T cell activation for Inflam-Monos (Fig. S3 B). Together, these data suggested that Isg15^+^ MAMs differentiated from Inflam-Monos might be important for the promotion of anti-androgen resistance in MycCaP-Bo model.

To test this directly, we used a mouse model with genetic ablation of CC chemokine receptor 2 (*Ccr2*), the major chemokine receptor mediating the recruitment of Inflam-Monos (Palframan et al., 2001; Getts et al., 2008). Similar to breast cancer models, MycCaP-Bo bone metastasis growth was significantly inhibited in syngeneic FVB *Ccr2*^*−/−*^ mice (Fig. 3, F and G; vehicle-treated groups [Veh]) deficient of Inflam-Monos as reported previously (Shi et al., 2011). In mice receiving enzalutamide treatment, the development of resistant tumors was also significantly inhibited (Fig. 3, F and G; Enz). Using TRAP staining, we determined that the density of osteoclasts located on bones surface adjacent to metastasis lesions was substantially reduced with pan-macrophage depletion by L-Clod, regardless of enzalutamide treatment (Fig. S3 I). In contrast, osteoclast abundance was not affected in monocyte-deficient *Ccr2*^−/−^ mice (Fig. S3 K). These data indicated that CCR2-recruited MAMs were important for anti-androgen resistance of MycCaP-Bo bone lesions, in an osteoclast independent manner.

CD169 has been recognized as the marker for bone marrow RTMs (Hashimoto et al., 2013). CD169^+^ RTMs were recently illustrated to contribute to tumor initiation of lung cancer (Casanova-Acebes et al., 2021). In the MycCaP-Bo model, RTMs were enriched for pathways of chemokine signaling, inflammatory response, and phagocytosis (Fig. S3 C). To test the role of CD169^+^ RTMs, we used transgenic mice expressing DTR under the control of CD169 promoter (CD169-DTR), in which CD169^+^ bone-resident macrophages can be depleted upon DT treatment compared with the control treatment with Glu^52^-DT (Ma et al., 2020). Consistently, macrophages associated with MycCaP-Bo bone lesions can be significantly depleted in CD169-DTR mice regardless of enzalutamide treatment (Fig. 3 H and Fig. S3, F and G). We further confirmed that CD106, another resident macrophage marker (Kaur et al., 2018), was also expressed by CD169^+^ macrophages, and CD106^+^CD169^+^ macrophages were efficiently depleted in CD169-DTR mice with DT treatment (Fig. S3 H). Similar to breast cancer bone metastasis models (Ma et al., 2020), this depletion did not affect MycCaP-Bo bone metastasis growth in vehicle-treated mice. In contrast, depletion of CD169^+^ macrophages synergistically inhibited MycCaP-Bo bone metastasis growth in combination with enzalutamide (coefficient of drug interaction = 0.59; Fig. 3, I and J). This indicated that CD169^+^ RTMs contributed to the development of enzalutamide resistance in MycCaP-Bo model. Furthermore, in resistant tumors, the ablation of CD169^+^ macrophages significantly inhibited continuous growth of resistant bone lesions (Fig. 3, K and L) indicating the importance of their continuous presence. Depletion of CD169^+^ bone-resident macrophages did not affect bone surface osteoclast density measured by TRAP staining (Fig. S3 J). Together, these data indicated that although CD169^+^ RTMs contribute minimally to bone metastasis growth, they are critical for enzalutamide resistance, again in an osteoclast independent manner. Collectively, our data indicated that both CD169^+^ RTMs and CCR2 recruited MDMs are critical for enzalutamide resistance of MycCaP-Bo bone lesions.

### Macrophage-induced FN1 expression promotes enzalutamide resistance

**Figure S4.**
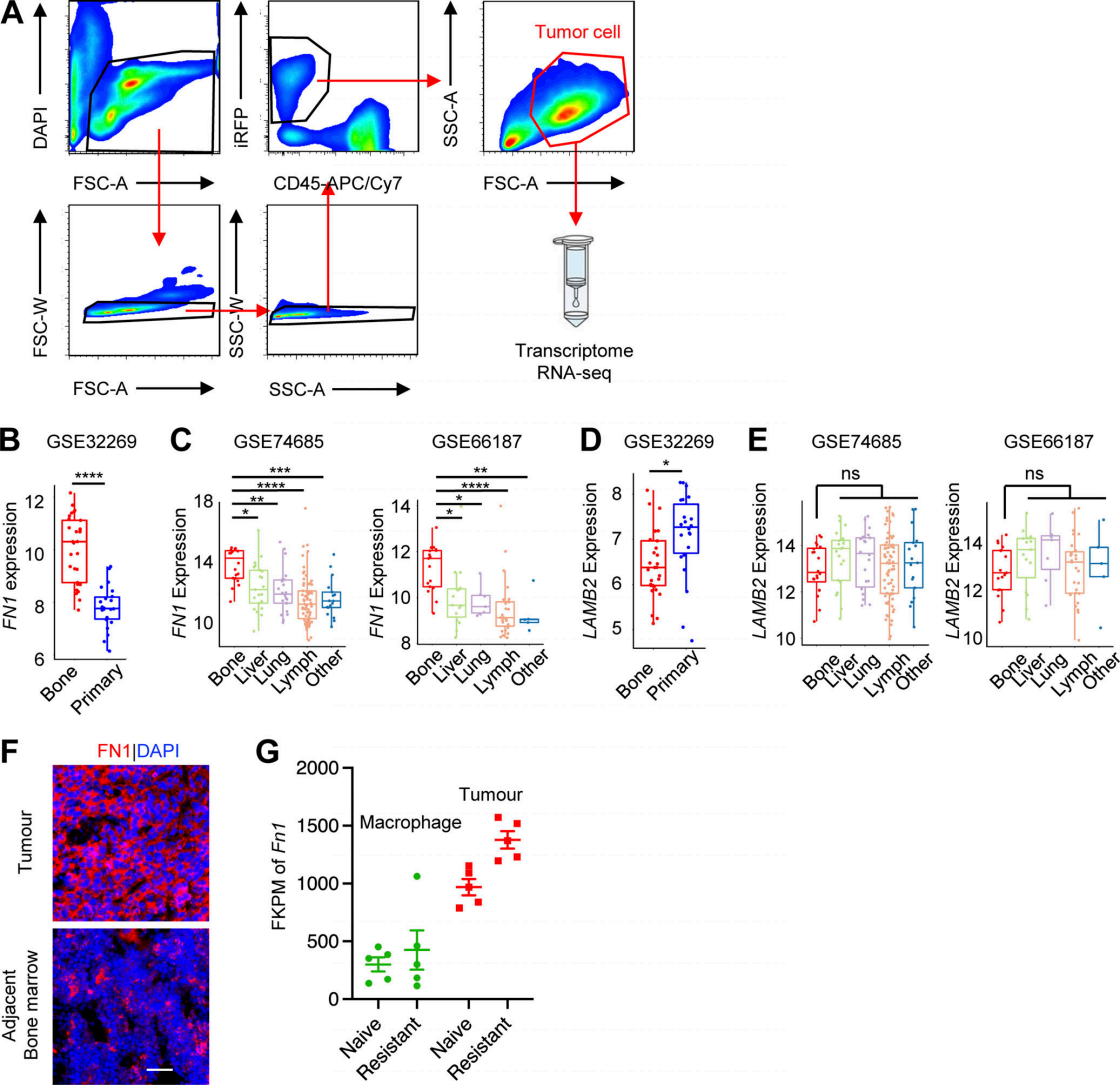
**Macrophages-mediated upregulation of FN1, but not LAMB2, in tumor cells is highly enriched in bone metastasis. (A)** Gating strategy of FACS sorting of tumor cells from bone metastasis lesions for transcriptome RNA-seq. **(B)** Expression of *FN1* in bone metastasis and primary PC in the indicated patient dataset. **(C)** Expression of *FN1* in bone metastasis and metastases from different organs in indicated patient datasets. **(D)** Expression of *LAMB2* in bone metastasis and primary PC in the indicated patient dataset. **(E)** Expression of *LAMB2* in bone metastasis and metastases from different organs in indicated patient datasets. **(F)** Representative image of FN1 IF staining in bone metastasis lesion and adjacent bone marrow. **(G)** Gene expression of *Fn1* (fragments per kilobase of transcript per million mapped reads [FPKM]) from RNA-seq of FACS purified MycCaP-Bo cells and macrophages from indicated tumors. *, P < 0.05; **, P < 0.01; ***, P < 0.001; ****, P < 0.0001, ns, not significant. Two-tailed unpaired Student’s *t* test was used in B and D, and ANOVA was used in C and E. Scale bar = 50 μm.

**Figure 4.**
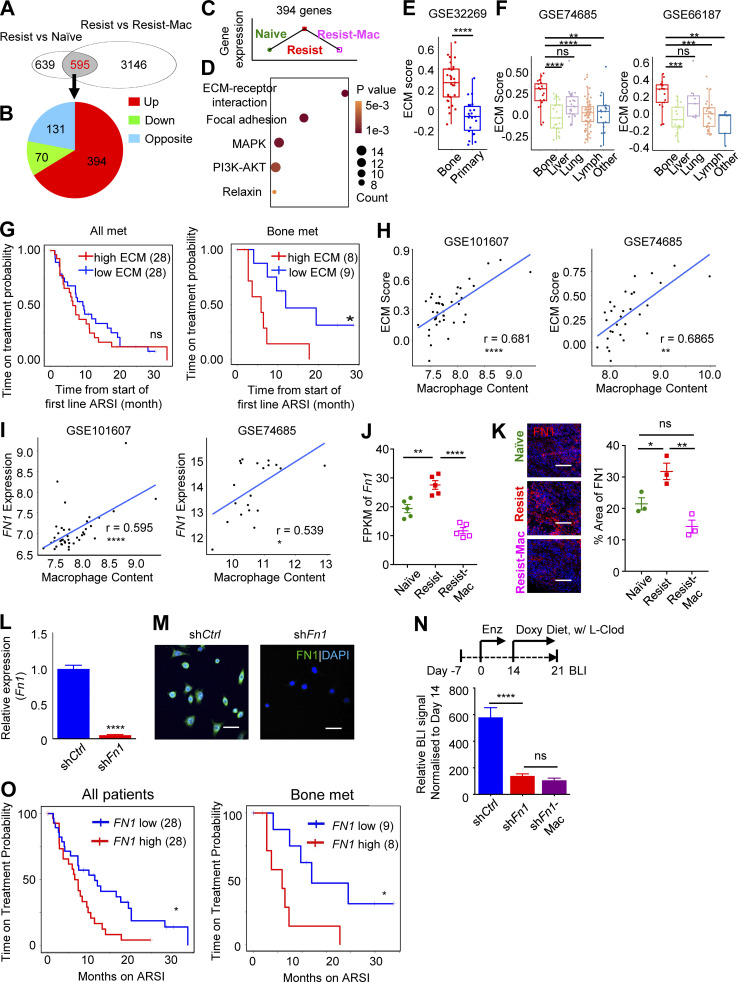
**Macrophage-induced tumor cell FN1 expression promotes enzalutamide resistance. (A)** Venn diagram showing numbers of differentially regulated genes from comparisons of RNA-seq transcriptome profiles of FACS-purified MycCaP-Bo cells from bone metastasis lesions with no treatment (Naive), enzalutamide treatment for 18 d (Resist), and enzalutamide treatment for 18 d plus macrophage depletion by L-Clod in the last 4 d (Resist-Mac). **(B)** Pie chart showing numbers of overlapping genes with indicated alterations from the two comparisons in A. **(C)** Schematic plot showing expression pattern of 394 genes upregulated in both comparisons from B. **(D)** Total five significantly enriched KEGG signaling pathways of the 394 upregulated genes from B. **(E)** ssGSEA estimation of KEGG ECM–receptor gene expression (ECM score) in bone metastasis and primary PC in patient dataset GSE32269. **(F)** ECM score in bone metastasis and metastases from different organs in indicated patient datasets. **(G)** Time on treatment (indicating resistance) probability of all patients (left) and patients with bone metastasis (right) who received anti-androgen therapy, with median expression of ECM score as cut-off. P value was calculated using Mantel–Cox test. **(H)** Correlation of ECM score with macrophage abundance in indicated bone metastasis datasets. **(I)** Correlation of *FN1* expression with macrophage abundance in indicated bone metastasis datasets. **(J)** Gene expression in FPKM from RNA-seq of FACS-purified MycCaP-Bo cells from indicated tumors. **(K)** Representative IF staining of MycCaP-Bo bone metastasis sections and quantification of FN1 protein. **(L)** Quantification of *Fn1* expression in doxycycline inducible *Fn1* knockdown MycCaP-Bo cells by real time PCR (*n* = 3). **(M)** Quantification of FN1 expression in doxycycline-inducible *Fn1* knockdown MycCaP-Bo cells by IF staining. **(N)** *Fn1* knockdown significantly inhibited resistant tumor growth in vivo*,* but not further affected by macrophage depletion using L-Clod, shown by quantification of BLI signals of bone metastasis on day 21 relative to day 14 derived from doxycycline-inducible sh*Ctrl* (blue), sh*Fn1* (red), or sh*Fn1*-Mac (purple) following the treatment scheme shown on top (*n* = 6∼8). **(O)** Time on treatment (indicating time to resistance) probability of all patients (left) and patients with bone metastasis (right) who received anti-androgen therapy, with median expression of FN1 as cut-off, showing high FN1 expression is significantly associated with anti-androgen resistance in mCRPC patients. Data are mean ± SEM in J–L and N; *, P < 0.05; **, P < 0.01; ***, P < 0.001; ****, P < 0.0001; ns, not significant. Two-tailed unpaired Student’s *t* test was used in E and L, and ANOVA was used in F, J, K, and N. Pearson correlation analysis was used in I. Mantel–Cox test was used in G and O. Scale bar = 100 μm.

To understand the mechanism of macrophage-induced enzalutamide resistance, bulk RNA-seq gene expression profiling was performed on MycCaP-Bo tumor cells FACS-purified based on their iRFP expression (>96% purity; Fig. S4 A) from naive tumors (vehicle), resistant tumors (Resist, 18 d enzalutamide) and resistant tumors with short-term depletion of macrophages (Resist-Mac, 18 d enzalutamide plus L-Clod in the last 4 d as described in Fig. 2 L). Using NOIseq analysis (Tarazona et al., 2012) and a threshold of fold change >1.5 and probability >0.8, 1,234 differentially regulated genes were identified to be associated with resistance (Resist vs. Naive) and 3,741 genes associated with macrophage depletion (Resist vs. Resist-Mac). Almost half of the resistance-associated genes are regulated by macrophages (595 out of 1,234) comparing both gene lists (Fig. 4 A). Among these overlapping genes, over three quarters of them were regulated in the same direction in both comparisons as shown in Fig. 4 A, which was consistent with the pro-resistance function of macrophages (Fig. 4 B). The 394 genes whose expression was upregulated in resistant tumors then downregulated upon macrophage depletion (expression pattern illustrated in Fig. 4 C) were most likely to be associated with macrophage-driven enzalutamide resistance. Pathway-enrichment analysis using DAVID (Huang et al., 2009) of these 394 genes identified 5 significantly enriched signaling pathways including: ECM–receptor interaction, focal adhesion, MAPK, phosphoinositide 3-kinases-Akt (PI3K/AKT), and Relaxin (Fig. 4 D).

ECM–receptor interaction was the most significantly enriched pathway among macrophage-regulated resistant genes (Fig. 4 D). This macrophage-regulated ECM gene expression program highly resembles the wound-healing response (Krzyszczyk et al., 2018; Olczyk et al., 2014). To determine whether ECM–receptor genes are relevant to patient bone-metastatic PC, we estimated the expression of this pathway using single sample gene set enrichment analysis (ssGSEA; Barbie et al., 2009) in patient gene expression datasets that contains both primary and bone-metastatic PC (Cai et al., 2013). Indeed, bone metastases have significantly higher ECM ssGSEA score compared with primary tumors, indicating the higher expression of genes in ECM–receptor interaction pathway (Fig. 4 E). Furthermore, these genes were also expressed at higher level in bone metastases compared with metastases in most of the other secondary organs (except lung) in two independent datasets that contain multiple metastases of PC (Haider et al., 2016; Zhang et al., 2015; Fig. 4 F). In the SU2C dataset, higher expression of ECM–receptor genes was significantly associated with anti-androgen resistance as measured by time on treatment in bone-metastatic PC samples but not in all metastasis samples combined (Fig. 4 G). Together these data strongly suggested that the ECM–receptor interaction pathway can be specifically involved in anti-androgen resistance of bone-metastatic PC.

The bulk RNA-seq data from MycCaP-Bo model indicated that a set of ECM–receptor genes were upregulated in enzalutamide-resistant tumors in a macrophage-dependent manner (Fig. 4, A–D). We reasoned that if this pathway is also regulated in a macrophage-dependent manner in patient bone metastases, their expression should show a positive correlation with macrophage abundance in patient datasets. Indeed, the ssGSEA scores of ECM–receptor interaction pathway genes are significantly correlated with macrophage abundance estimated by ImSig in two independent PC bone metastasis datasets (Cai et al., 2013; Ylitalo et al., 2017; Fig. 4 H). Among all the ECM genes that followed the expression pattern as shown in Fig. 4 C, laminin subunit beta-2 (*LAMB2*), and *FN1* were significantly correlated with macrophage abundance in more than two patient datasets (Fig. 4 I and data not shown). Between these two genes, *FN1*, but not *LAMB2*, was expressed in a higher level in bone metastasis comparing to primary tumor and metastases in other organs using human datasets (Fig. S4, B–E), suggesting a specific involvement of FN1 in bone-metastatic PC. Consistently, in the MycCaP-Bo bone metastasis model, FN1 expression, at both mRNA and protein level, increased in resistant tumors compared with naive tumors and decreased upon macrophage ablation (Fig. 4, J and K). In addition, FN1 expression was significantly higher in tumor cells compared with that in macrophages (Fig. S4, F and G). Therefore, we hypothesized that FN1 in tumor cells may be a key macrophage-regulated ECM gene that promoted enzalutamide resistance in bone-metastatic PC. To test this, we generated MycCaP-Bo cells that expressed doxycycline inducible shRNA (sh*Fn1*) knocking down *Fn1* expression at both mRNA and protein levels compared with control shRNA (sh*Ctrl*; Fig. 4, L and M). This inducible *Fn1* knockdown significantly inhibited enzalutamide resistance of MycCaP-Bo bone metastasis in vivo. Furthermore, the resistance cannot be further inhibited with macrophages ablation (Fig. 4 N), indicating a critical role of tumor cell–derived FN1 in driving resistance downstream of macrophages. Consistently, in the SU2C dataset, the higher *FN1* expression with a median threshold was significantly associated with therapy resistance as measured by time on treatment in all 56 patients with different metastasis combined, which was even more significant in patients with bone metastasis (Fig. 4 O). Together, these data indicated that macrophage-induced tumor cell FN1 expression significantly promoted anti-androgen resistance of bone-metastatic PC.

### Macrophage-induced integrin a5 (ITGA5) expression promotes enzalutamide resistance

**Figure 5.**
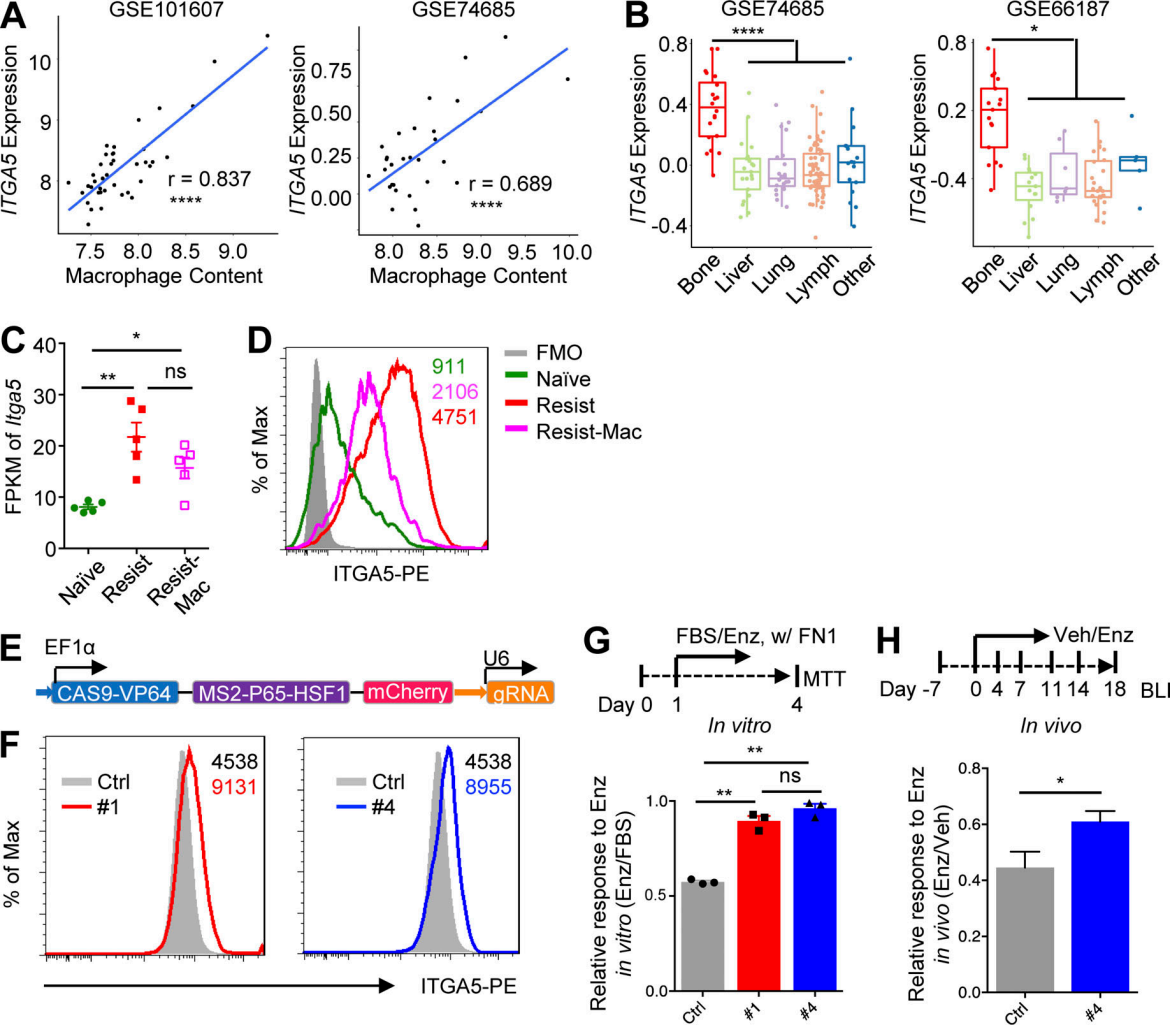
**Macrophage-induced integrin a5 (ITGA5) expression promotes enzalutamide resistance. (A)** Correlation of *ITGA5* expression with macrophage abundance in indicated bone metastasis datasets. **(B)** Expression of *ITGA5* in bone metastasis and metastases from different organs in indicated patient datasets. **(C)** Expression of *Itga5* in FPKM of MycCaP-Bo cells FACS-purified from indicated in vivo bone metastasis. **(D)** Representative histogram of ITGA5 expression on MycCaP-Bo cells FACS-purified from indicated in vivo bone metastasis. Number indicates mean fluorescent intensity. FMO, fluorescent minus one; negative control for flow cytometry staining. **(E)** Schematic showing key elements in the UniSAM vector. **(F)** Flow histogram of ITGA5 expression in control (Ctrl), and *Itga5* overexpressing MycCaP-Bo cells clone 1 (#1 sgRNA, left) and clone 4 (#4 sgRNA, right); number indicating MFI. **(G)** In vitro response of control (Ctrl) and cells overexpressing *Itga5* (#1, #4) to enzalutamide treatment in the presence of FN1 (1 μg/ml). Response was defined by relative growth of indicated cells with enzalutamide (1 μM) over vehicle treatment (*n* = 3). **(H)** High expression of *Itga5* promotes enzalutamide resistance in vivo shown by relative growth of control (Ctrl) and cells overexpressing *Itga5* (#4) with enzalutamide treatment versus vehicle, shown as relative BLI signal of enzalutamide treatment over vehicle treatment on day 18. Data are mean ± SEM in C, G, and H; *, P < 0.05; **, P < 0.01; ****, P < 0.0001; ns, not significant. Pearson correlation analysis was used in A, ANOVA was used in B, C, and G, and two-tailed unpaired Student’s *t* test was used in H.

After identified that FN1 was the key ECM gene that drove anti-androgen resistance of bone-metastatic PC, we set out to identify the key ECM receptor gene. Among all the ECM receptor genes that followed the expression pattern as shown in Fig. 4 C, *ITGA5*, a receptor of FN1 (Eble and Niland, 2019), was strongly correlated with macrophage abundance in multiple human PC bone metastasis datasets (Ylitalo et al., 2017; Haider et al., 2016; Fig. 5 A). The expression level of *ITGA5* was also significantly higher in bone metastases compared with metastases from other organs (Fig. 5 B). In MycCaP-Bo bone lesions, mRNA level of *Itga5* was upregulated in enzalutamide-resistant tumors (Resist) compared with naive tumors (Naive) and downregulated with macrophage depletion (Resist-Mac; Fig. 5 C). This was translated into alteration at protein level as determined by FACS analysis (Fig. 5 D). Together, these data indicated that *Itga5* was indeed highly expressed in PC bone disease in a macrophage-dependent manner suggesting its potential role in macrophage and FN1 promoted anti-androgen resistance.

To test this directly, we upregulated the expression of endogenous *Itga5* in MycCaP-Bo cells using the UniSAM vector, a mutated Cas9-VP64 system (Fidanza et al., 2017; Fig. 5 E). Two different gRNAs recognizing the promoter region designed using an online tool (https://www.benchling.com/crispr) significantly upregulated ITGA5 expression compared with control gRNA (Ctrl) to about twofold in vitro, which was comparable to the level of change in vivo with macrophage depletion (Fig. 5 F). This led to increased resistance to enzalutamide in vitro in the presence of FN1 (Fig. 5 G) and in vivo compared with control cells (Fig. 5 H). Together, these data indicated that macrophage-induced tumor cell ITGA5 expression can promote enzalutamide resistance of bone-metastatic PC.

### Macrophage-derived activin-A–induced FN1-ITGA5 axis in bone-metastatic PC

**Figure 6.**
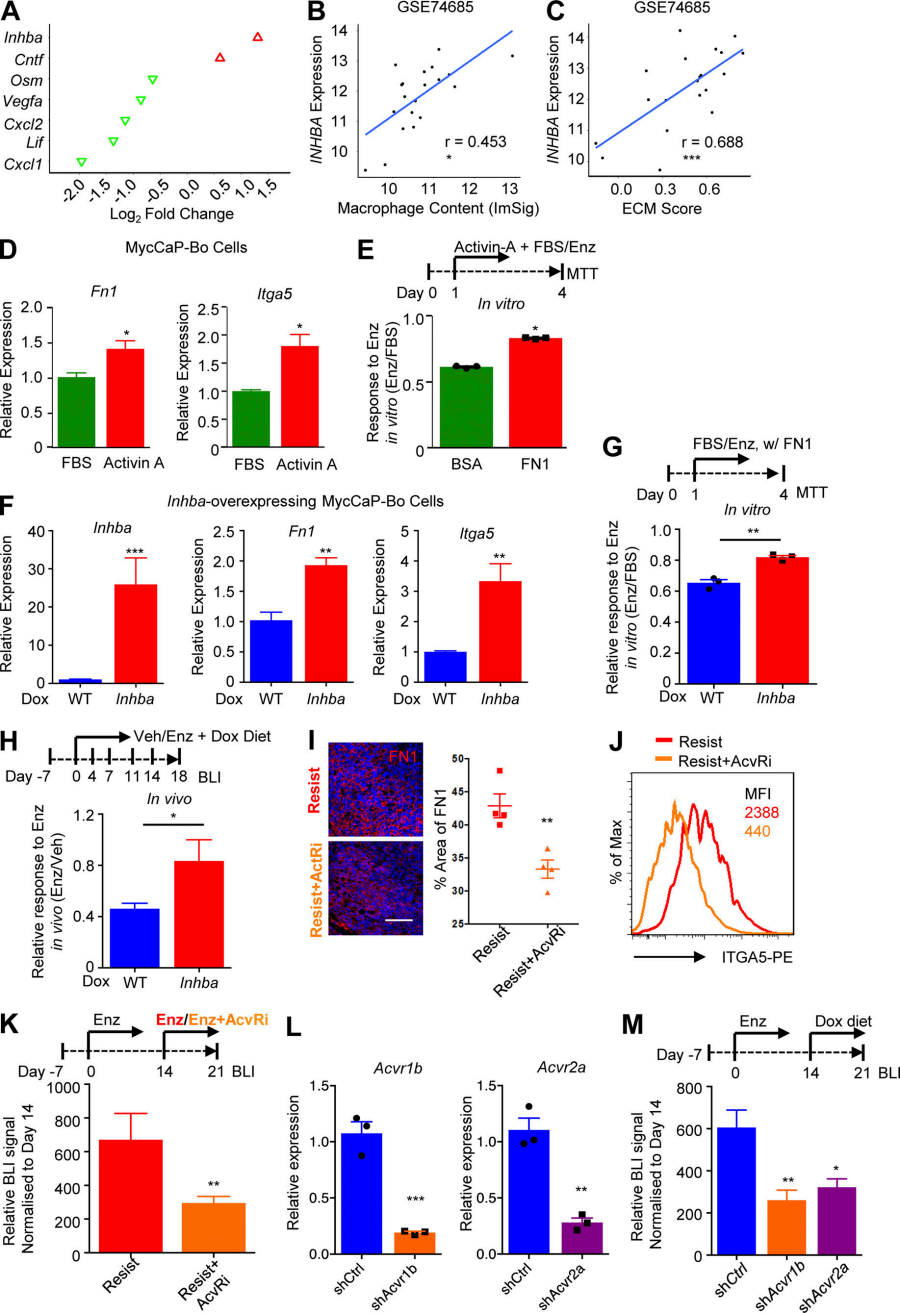
**Macrophage-derived activin-A induction of FN1-ITGA5 axis in bone-metastatic PC. (A)** Relative expression of all significantly altered cytokine genes of monocytes and macrophages associated with in vivo enzalutamide-resistant MycCaP-Bo bone metastasis compared with those associated with naive tumors as defined in Fig. 4 A. Red and green indicates up- and downregulated in resistance-associated monocytes/macrophages, respectively. **(B)** Correlation of *INHBA* expression with macrophage content in indicated patient bone metastasis dataset. **(C)** Correlation of *INHBA* expression with ECM score in indicated patient bone metastasis dataset. **(D)** Relative expression of *Fn1* and *Itga5* in MycCaP-Bo cells treated with activin-A quantified by qPCR (*n* = 4). **(E)** Response of MycCaP-Bo cells treated with activin-A (20 ng/ml) to enzalutamide treatment in the presence of BSA (1%) or FN1 (1 μg/ml). Response was defined by relative growth of indicated cells with enzalutamide (1 μM) to FBS-cultured cells (*n* = 3). **(F)** Relative expression of *Inhba*, *Fn1*, and *Itga5* in *Inhba* doxycycline-inducible overexpressing MycCaP-Bo cells treated with doxycycline quantified by qPCR (*n* = 3). **(G)** Response of control (WT) cells and cells with inducible overexpression of *Inhba* to enzalutamide treatment in the presence of fibronectin in vitro. Response was defined by relative growth of indicated cells with enzalutamide (1 μM) to FBS-cultured cells (*n* = 3). **(H)** Overexpression of *Inhba* drives resistance in vivo indicated by enzalutamide response of control (WT) and cells with inducible overexpression of *Inhba* following the treatments shown in the diagram on top. Data shown as the relative growth of indicated cells with enzalutamide to vehicle on day 18 (*n* = 10). **(I)** Representative images and quantification of FN1 staining in MycCaP-Bo bone metastasis with indicated treatment. **(J)** Flow cytometry histogram of ITGA5 expression on tumor cells in MycCaP-Bo bone metastasis with indicated treatment as shown in the top diagram of J); number indicates MFI. **(K)** Activin receptor signaling is critical for enzalutamide resistance shown by representative growth of resistant MycCaP-Bo bone metastasis following indicated treatment (*n* = 12). **(L)** Quantification of *Acvr1b* and *Acvr2a* expression in indicated doxycycline-inducible knockdown MycCaP-Bo cells by qPCR (*n* = 3). **(M)** Activin receptor is critical for enzalutamide resistance in vivo indicated by quantification of BLI signals on day 21 relative to day 14 of bone metastasis of doxycycline-inducible sh*Ctrl,* sh*Acvr1b*, or sh*Acvr2a* MycCaP-Bo cells following the treatment scheme shown on top (*n* = 6). Data are mean ± SEM; *, P < 0.05; **, P < 0.01; ***, P < 0.001. Two-tailed unpaired Student’s *t* test. Pearson correlation analysis was used in B and C. Scale bar = 100 μm.

**Figure S5.**
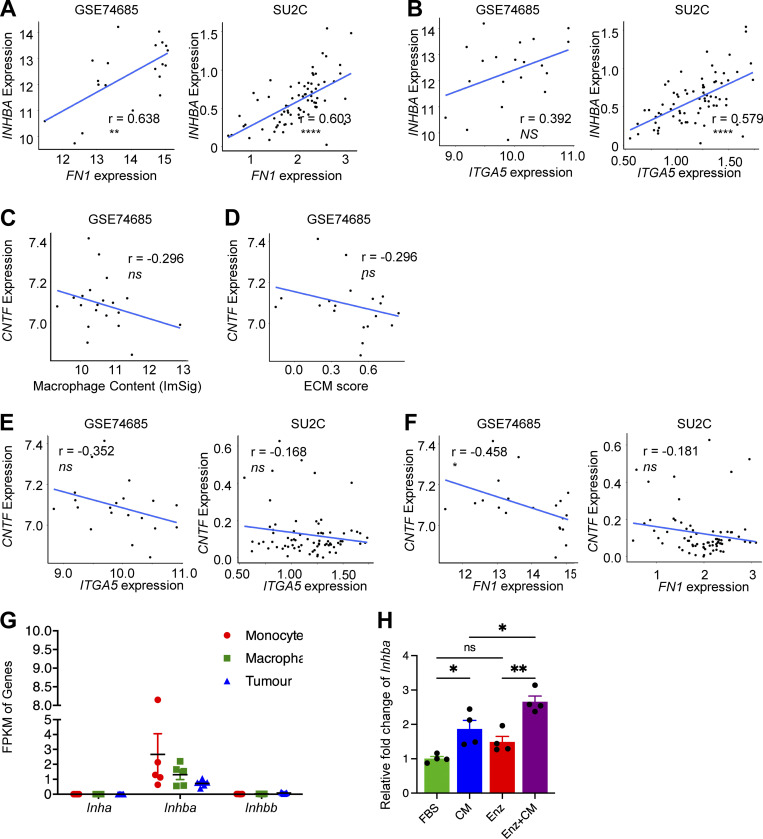
***INHBA*, but not *CNTF*, correlated with macrophage content. (A)** Correlation of *INHBA* with *FN1* in independent patient datasets. **(B)** Correlation of *INHBA* with *ITGA5* in independent patient datasets. **(C)** Correlation of *CNTF* with macrophage content in independent patient datasets. **(D)** Correlation of *CNTF* with ECM score in independent patient datasets. **(E)** Correlation of *CNTF* with *ITGA5* in independent patient datasets. **(F)** Correlation of *CNTF* with *FN1* in independent patient datasets. **(G)** FPKM of three *Inhibin* genes in FACS-purified monocyte, macrophage, and tumor cells from enzalutamide-resistant bone metastasis of MycCaP-Bo cells as defined in Fig. 4 A. **(H)** Relative expression of *Inhba* in bone marrow–derived macrophages cultured alone (Ctrl), treated with conditional medium of MycCaP-Bo (CM), enzalutamide (1 μM, Enz) or conditioned medium of MycCaP-Bo cells and enzalutamide together (CM+Enz) quantified by qPCR (*n* = 4). Data are mean ± SEM; *, P < 0.05; **, P < 0.01; ns, not significant. ANOVA test was used in H, and Pearson correlation analysis was used in A–F.

To understand the mechanism of how macrophages induced FN1-ITGA5 expression in bone-metastatic PC, RNA sequencing gene expression profiling was performed using FACS purified monocytes (CD45^+^CD11b^+^Ly-6C^hi^CCR2^+^) and macrophages (CD45^+^CD11b^+^Ly-6C^−^Ly-6G^−^F4/80^+^SSC^low^; gating strategy specified in Fig. S2 F; purity >96%) from naive tumors (vehicle) and resistant tumors (enzalutamide 18 d) as described in Fig. 4 A. Using NOIseq analysis (Tarazona et al., 2012) and a threshold of fold change >1.5, probability >0.8, 297 and 560 differentially regulated genes were identified to be associated with resistance (resist vs. naive) in monocytes and macrophages, respectively (data not shown). Among these genes, we decided to focus on cytokines as they are the major modulators of the tumor microenvironment. Among all the seven differentially expressed cytokines, inhibin beta A (*Inhba*) and ciliary neurotrophic factor (*Cntf*) were upregulated in monocytes associated with resistant tumors in MycCaP-Bo model (Fig. 6 A). In patient bone metastasis datasets, expression of *INHBA*, but not *CNTF*, was significantly correlated with macrophage abundance, ECM–receptor pathway score and expression level of *FN1* and *ITGA5* (Fig. 6, B and C; and Fig. S5, A–F), suggesting the potential role of *IHNBA* in mediating macrophage-regulated expression of the ECM–receptor pathway, and FN1 and ITGA5 genes. INHBA forms biologically active hetero- or homo-dimeric protein complexes of inhibin and activin with other two family member genes: inhibin α (*INHA*) and inhibin β β (*INHBB*; Burger and Igarashi, 1988). In our RNA-seq data, *Inha* and *Inhbb* were barely expressed by either tumor cells, monocytes, or macrophages from MycCaP-Bo tumors (Fig. S5 G). This suggested that the homodimer of INHBA, activin A, can be the main macrophage-derived cytokine that drove ECM–receptor gene expression in bone-metastatic PC.

To test this, we treated MycCaP-Bo cells with activin A in vitro. Supporting our hypothesis, activin A induced the expression of both *Fn1* and *Itga5* (Fig. 6 D) and promoted enzalutamide resistance growth of MycCaP-Bo cells in vitro in the presence of FN1 (Fig. 6 E). To test whether activin A can drive resistance growth in vivo, we generated MycCaP-Bo cells with doxycycline-inducible expression of *Inhba*. As expected, doxycycline significantly induced *Inhba* gene expression in these cells and subsequently upregulated the expression of *Fn1* and *Itga5* in vitro (Fig. 6 F). Importantly, this *Inhba* upregulation significantly promoted enzalutamide resistance growth of MycCaP-Bo cells in vitro in the presence of FN1 (Fig. 6 G) and MycCaP-Bo bone metastasis in vivo (Fig. 6 H), indicating its important role in driving resistance. Activin A signals through activin receptors AcvRIIA/AcvRIIB, type II serine threonine kinase receptors, together with ALK4 (Massague, 1996). SB-505124, a selective inhibitor against activin A receptors (Marini et al., 2018), significantly inhibited FN1 accumulation (Fig. 6 I) and ITGA5 expression (Fig. 6 J) of MycCaP-Bo bone metastasis in vivo. This led to a significant inhibition of enzalutamide resistance growth of these bone metastasis lesions (Fig. 6 K). To determine whether tumor cell activin receptor signaling is important in enzalutamide resistance in vivo, we generated MycCaP-Bo cells expressing doxycycline-inducible shRNA targeting *Acvr1b* and *Acvr2a*, respectively. Specific knockdown of either *Acvr1b* or *Acvr2a* significantly inhibited the growth of enzalutamide resistant bone lesions in vivo (Fig. 6, L and M). Together, these data indicated that activin A–receptor was the major cytokine signaling that drove anti-androgen resistance of MycCaP-Bo bone-metastatic PC in vivo.

Bone marrow–derived macrophages expressed increased level of *Inhba* upon the treatment of conditional medium of MycCaP-Bo cells and enzalutamide in vitro, such increase was further induced when conditional medium and enzalutamide were combined (Fig. S5 H). This suggests that the macrophage *Inhba* can be induced by both enzalutamide and tumor cell secreted factors.

### Enzalutamide resistance of bone-metastatic PC can be blocked by SRC-specific inhibitor

**Figure 7.**
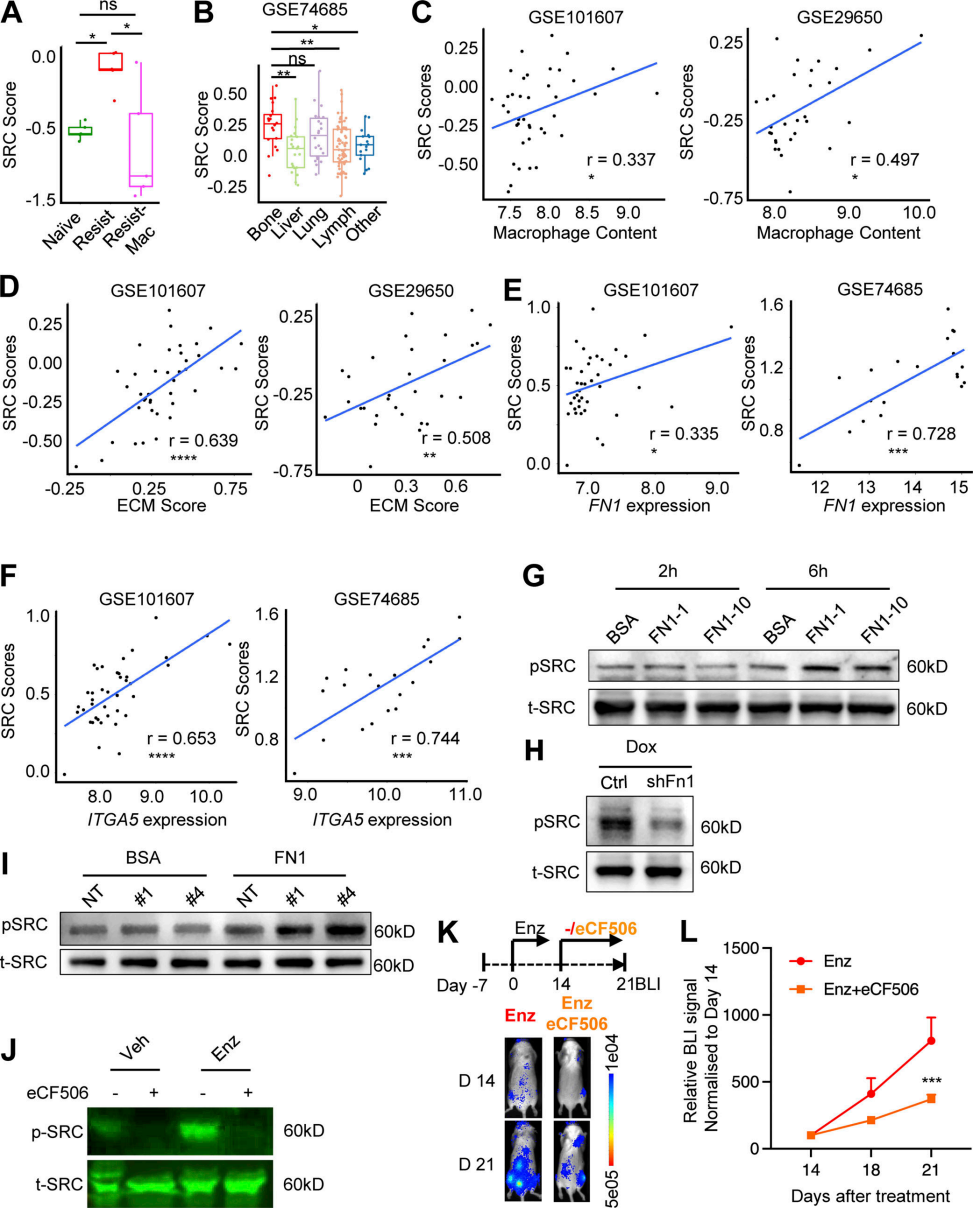
**Enzalutamide resistance of bone-metastatic PC can be blocked by SRC-specific inhibitor. (A)** Src activity estimated using Src score (see Materials and methods) in RNA-seq data of FACS-purified MycCaP-Bo tumor cells from indicated disease stage/treatment (as described in Fig. 4 A). **(B)** Src score in patient bone metastasis and metastases from other organs. **(C)** Correlation of Src score with macrophage abundance in patient bone metastasis datasets. **(D)** Correlation of Src score with ECM score in patient bone metastasis datasets. **(E)** Correlation of Src score with *FN1* expression in patient bone metastasis datasets. **(F)** Correlation of Src score with *ITGA5* expression in patient bone metastasis datasets. **(G)** Immunoblot showing the level of phosphorylated SRC (pSRC) and total SRC (t-SRC) in MycCaP-Bo cells seeded in wells precoated with 1% BSA (BSA), 1 μg/ml FN1 (FN1-1), and 10 μg/ml FN1 (FN1-10) for indicated time. **(H)** Immunoblotting showing the level of pSRC and t-SRC in modified MycCaP-Bo cells with doxycycline-induced expression of control shRNA or shRNA-targeting Fn1. The cells were treated with doxycycline (500 ng/ml) for 4 d. **(I)** Immunoblotting showing the level of pSRC and t-SRC in control MycCaP-Bo cells (Ctrl), MycCaP-Bo cells overexpressing ITGA5 clone 1 (#1) and clone 4 (#4) seeded in wells precoated with 1% BSA (BSA) or 1 μg/ml FN1 (FN1) for 6 h before sample harvest. **(J)** Immunoblotting showing the level of pSRC and t-SRC in in vivo MycCaP-Bo bone metastasis samples with indicated treatments. **(K)** Representative BLI images of resistant MycCaP-Bo bone metastasis following eCF506 treatment. **(L)** BLI quantification of resistant MycCaP-Bo bone metastasis following eCF506 treatment (*n* = 8∼10). Data are mean ± SEM; *, P < 0.05; **, P < 0.01; ns, not significant. ANOVA was used in A and B, two-tailed unpaired Student’s *t* test was used for L, and Pearson correlation analysis was used in C–F. Source data are available for this figure: SourceData F7.

Focal adhesion pathway is the major pathway that mediates downstream signaling of ECM–receptor interaction (Seguin et al., 2015) and the second most significantly enriched pathway in macrophage-dependent resistance-associated genes in RNA-seq data of MycCaP-Bo bone lesions (Fig. 4 D). This suggested that focal adhesion pathway may be important to FN1-ITGA5–induced anti-androgen resistance. Tyrosine kinase Src is the major activator in the focal adhesion pathway (Guo and Giancotti, 2004; Seguin et al., 2015), and Src activity has been shown to be upregulated in castration-resistant PC samples and involved in androgen-independent growth (Varkaris et al., 2014; Tatarov et al., 2009). To determine Src activity in bone-metastatic PC, we generated an ssGSEA-based Src activity score using an Src-induced gene expression signature in primary prostate epithelial cells (GSE37428). Analysis in the RNA-seq data of MycCaP-Bo cells purified from in vivo bone metastasis lesions illustrated a significant increase of Src activity in resistant tumors compared with naive tumors which was downregulated upon macrophage depletion (Fig. 7 A). In patient datasets, Src activity was significantly higher in bone metastases compared with metastases in other secondary organs (except lung; Fig. 7 B). Furthermore, Src activity was also significantly correlated with macrophage abundance, ECM–receptor pathway score, and expression of *FN1* and *ITGA5* in human bone metastasis datasets (Fig. 7, C–F).

To further confirm whether FN1-ITGA5 interaction leads to SRC activation, we measured SRC phosphorylation in MycCaP-Bo cells using Western blot. SRC phosphorylation was upregulated in MycCaP-Bo cells after 6 h of seeding in FN1-coated plates compared with control (Fig. 7 G). Consistently, knock-down of *Fn1* in MycCaP-Bo cells reduced SRC phosphorylation compared with control cells (Fig. 7 H). Overexpression of ITGA5 in MycCaP-Bo cells further increased SRC phosphorylation in presence of FN1 coating, compared with control MycCaP-Bo cells (Fig. 7 I). Together, our data suggested that Src activity in patient PC bone disease can be regulated by INHBA-induced FN1-ITGA5 interaction.

Previous SRC targeting reagents had minimal success in clinic partly due to their broad activity against multiple kinases and associated toxicity (Puls et al., 2011; Zhang and Yu, 2012). A novel orally bioavailable compound, eCF506, was recently discovered to only inhibit Src family kinases (SFKs), displaying superior selectivity and lower off-target effects than any other Src/Abl inhibitor either approved or in clinical development (Fraser et al., 2016). The in vivo potency and unique selectivity profile of eCF506, combined with its good PK properties (Fraser et al., 2016), makes it an ideal tool for preclinical research and a suitable candidate for clinical development. Using MycCaP-Bo bone metastasis model, we generated enzalutamide-resistant tumors with 14 d of enzalutamide treatment to further test the effect of SFK-specific inhibition using eCF506. In these tumors, as expected, SRC phosphorylation was significantly increased compared with naive tumors, which was completely inhibited by eCF506 (Fig. 7 J). This treatment significantly inhibited enzalutamide resistance compared with vehicle control (Fig. 7, K and L). Together, these data indicated that macrophage-induced Src activity is critical for anti-androgen resistance of bone-metastatic PC and eCF506 may offer a promising therapeutic agent to treat this deadly disease.

## Discussion

Despite many progresses in understanding of ADT resistance using primary tumor models, the mechanism by which metastasis microenvironment promotes the development of anti-androgen resistance of metastatic PC is largely unknown. In the current study, we developed a novel androgen-dependent bone-metastatic PC model in immune-competent syngeneic mice using intra-cardiac injection of MycCaP-Bo cells. Our model showed a mixed osteogenic and osteolytic pathology, and significant amounts of macrophage infiltration, resembling patient diseases. In the current study, upon enzalutamide treatment, the MycCaP-Bo model mimicked the naive-responsive resistance disease progression in patients. This allows the differentiation between the processes of metastasis and anti-androgen resistance and investigation of the role of metastasis microenvironment in enzalutamide resistance in vivo.

Using the MycCaP-Bo model, the current study illustrated a novel PC cell non-autonomous mechanism of anti-androgen resistance. This involved a wound-healing–like response of ECM and receptor gene expression in PC cells induced by macrophage-derived activin A, providing a novel mechanistic link between wound-healing response and hormone resistance in metastatic disease. Originally identified as regulator of follicle-stimulating hormone, activin A is a TGF-β family cytokine and plays an important role in promoting wound healing and scar formation (Cangkrama et al., 2020). Activin A has been shown to potently inhibit the growth of normal prostate and some PC cells (McPherson et al., 1997; Dowling and Risbridger, 2000). In contrast, circulating level of activin A was associated with bone diseases of breast cancers and PCs (Leto et al., 2006). Activin A–NF-κB signaling has recently been shown to promote PC metastasis through induction of cancer stem cells (Chen et al., 2020; Gold and Risbridger, 2012). Our data illustrated a novel mechanism of activin A in anti-androgen resistance linking macrophages with upregulation of FN1-ITGA5 signaling axis in cancer cells. Consistent with our results, recent studies using scRNA-seq of metastatic PC patient samples showed that enzalutamide-exposed PC cells robustly upregulated gene sets downstream of TGF-β signaling rather than enriched for tumor clones carrying resistant mutations (He et al., 2021). Together these data suggested an interesting new model that a wound-healing response induced by macrophages promotes hormone resistance before significantly accumulation of genetic alterations and provides another example of tumor cell hijacking normal physiological processes to achieve their malignant purpose.

A previous study reported that IL-23 derived from polymorphonuclear myeloid–derived suppressor cells (neutrophils) activated AR signaling to promote ADT resistance in in vivo model of primary PC (Calcinotto et al., 2018). Conditioned medium from these MDSCs or recombinant IL-23 promoted PC cell resistance in vitro (Calcinotto et al., 2018). In the MycCaP-Bo model, Ly-6G^+^ neutrophils were another major myeloid cell population. However, Ly-6G^+^ depletion Ab showed no effect on the anti-androgen resistance in vivo (Fig. S2 L). In addition, neither recombinant IL-23 nor conditioned medium from bone marrow MDSC was able to promote enzalutamide resistance in vitro (Fig. S2, M and N). These data may reflect the differences between the models used, and/or the differences between bone metastasis versus primary tumors. The latter further highlighted the unique mechanism of bone-metastatic PC relying on the interaction with macrophages.

Our data indicated that macrophage abundance and ECM–receptor gene expression are significantly increased in bone metastasis compared with primary tumor and soft tissue metastases and associated with anti-androgen resistance and poor survival in patient datasets. This indicated that this mechanism can be particularly important for metastatic PC in bone. Among the ECM–receptor genes, our data illustrated that FN1-ITGA5 axis played a critical role in the promotion of enzalutamide resistance. FN1 is a core component of the tumor matrisome and was upregulated in metastatic PC patients compared with normal and benign prostatic hyperplasia samples (Konac et al., 2017). As the major receptor class of ECM, integrins are critical for cells to respond to matrix alterations. Integrin β1 and αV integrin subunits have been shown to mediate resistance to chemotherapy, radiation therapy, and targeted therapies (Cooper and Giancotti, 2019). Our results showed that the expressions of FN1 and ITGA5 are strongly correlated with macrophage abundance in multiple patient datasets. Together, these data strongly argued for a novel and potent role of macrophage-induced FN1-ITGA5 signaling in anti-androgen resistance of bone-metastatic PC.

SFKs are the major signal transducer of canonical integrin signaling pathways (Seguin et al., 2015). The current study illustrated for the first time that upregulated Src activity in enzalutamide-resistant MycCaP-Bo bone lesions in vivo is dependent on macrophages. This is consistent with a strong correlation between SRC activity score and macrophage abundance in bone-metastatic PC transcriptome datasets. Although Src activity has long been noticed in mCRPC (Vlaeminck-Guillem et al., 2014; Varkaris et al., 2014), previous clinical trials targeting SFKs were rather disappointing (Araujo et al., 2013; Antonarakis et al., 2013, Lara et al., 2009). While SFKs inhibition appears to be an obvious and promising anticancer strategy, SFK signaling is complex and resistance to SFK inhibitors can result from multiple genetic, epigenetic, and adaptive post-translational signaling mechanisms (Zhang and Yu, 2012). To date, all the completed clinical trials of SFK inhibitors in solid tumors have been performed using monotherapy in unselected patients examining short-term endpoints. Thus, the identification of biomarkers to stratify patients with activated SFK signaling, longer term disease progression and survival endpoints, and rational drug combination strategies are likely to support more effective clinical trial design. While a major reason for this lack of clinical success is likely associated with poor clinical trial design, it is important to note that all previous SRC inhibitors tested in the clinic also target other kinases which induce toxicity or inhibition of the immune system and confound optimal trial design (Rivera-Torres and San José, 2019). For example, dasatinib has been shown to inhibit multiple tyrosine kinases in the sub-nanomolar range (Karaman et al., 2008) and has been associated with substantial undesired side effects (Kreutzman et al., 2017; Yang et al., 2015). eCF506 is a novel SFK inhibitor that has superior selectivity and high potency specifically against SRC, YES, and LYN (Temps et al., 2021). In contrast to other SRC inhibitors such as dasatinib, eCF506 does not inhibit ABL and inhibits other Src family members with 10 to 100 lower potency, including BLK and HCK (Fraser et al., 2016; Temps et al., 2021), which are thought to be important for immune cell maturation and activity (Byeon et al., 2012). Our data demonstrated that specific SRC inhibition using eCF506 potently inhibited resistant growth in vivo. This indicates that specific targeting of SRC can be a valid approach to treat anti-androgen resistance of metastatic PC, at least in a subset of patients with high macrophage infiltration and/or ECM gene expression. This opens an exciting opportunity to evaluate the therapeutic potential of eCF506 in clinical trials in patients.

## Materials and methods

### Generation of MycCaP-Bo cell line and modified sublines

#### MycCaP-Bo cells

pLEGFP-N1-iRFP-2A-Fluc plasmid was derived from pLEGFP-N1 vector by inserting fluorescence protein iRFP (Filonov et al., 2011) and firefly luciferase into HindIII and MluI site. 2A was used as linker between two genes for co-expression (Szymczak-Workman et al., 2012). Virus containing target plasmid was generated from 72 h culture supernatant of phoenix cells transfected with 5 μg pLEGFP-N1-iRFP-2A-Fluc, 2 μg PAX2, and 2 μg VSVg.

MycCaP cells were infected with virus mentioned above, selected by antibiotics G418 (1 mg/ml; Sigma-Aldrich) and FACS sorted for iRFP^+^ cells. The selected cancer cells were intracardiacally injected into WT FVB/N males to develop bone metastasis three times for selection of bone-metastatic tumor cells and named as MycCaP-Bo cells.

#### Inducible knockdown MycCaP-Bo cells and inducible Inhba overexpression MycCaP-Bo cells

The lentiviral LT3CEPIR vectors were generated by switching the GFP cassette in (LT3GEPIR) miR-E–based expression vector with a mCherry cassette (Fellmann et al., 2013; Mu et al., 2017).

The sequences of shRNA hairpins are as follows: shCtrl: 5′-TGC​TGT​TGA​CAG​TGA​GCG​CAG​GAA​TTA​TAA​TGC​TTA​TCT​ATA​GTG​AAG​CCA​CAG​ATG​TAT​AGA​TAA​GCA​TTA​TAA​TTC​CTA​TGC​CTA​CTG​CCT​CGG​A-3′; shFn1: 5′-TGC​TGT​TGA​CAG​TGA​GCG​AAA​AGA​CAA​GTG​TTT​TAA​TAA​ATA​GTG​AAG​CCA​CAG​ATG​TAT​TTA​TTA​AAA​CAC​TTG​TCT​TTC​TGC​CTA​CTG​CCT​CGG​A-3′; shAcvr1b: 5′-TGC​TGT​TGA​CAG​TGA​GCG​ACG​AGC​TGA​ATA​TGG​TGT​TTA​ATA​GTG​AAG​CCA​CAG​ATG​TAT​TAA​ACA​CCA​TAT​TCA​GCT​CGG​TGC​CTA​CTG​CCT​CGG​A-3′; shAcvr2a: 5′-TGC​TGT​TGA​CAG​TGA​GCG​ACA​GGA​AGT​TGT​TGT​GCA​TAA​ATA​GTG​AAG​CCA​CAG​ATG​TAT​TTA​TGC​ACA​ACA​ACT​TCC​TGC​TGC​CTA​CTG​CCT​CGG​A-3′.

Murine *Inhba* gene coding region was cloned into pCW57.1 (#41393; Addgene) using gateway cloning methods.

To generate lentivirus particles expressing shRNAs or *Inhba*, 1.2 million HEK-293T cells were seeded into a 6-well plate, followed by next day transfection of corresponding plasmids (2 μg) together with psPAX2 packaging plasmid (2 μg) and pVSV-G plasmid (1 μg) using Fugene HD transfection reagents (E2311;Promega). The medium was replaced by fresh medium 20 h later and harvest the medium containing lentivirus particles 48 h later.

100,000 MycCaP-Bo cells were seeded into a 6-well plate. On the next day, the medium was removed and replaced with medium containing 50% medium containing lentivirus, 50% fresh medium, and 8 μg/ml polybrene. The selection was started 48 h after the virus transduction by 5 μg/ml puromycin for 5 d. Inducible knockdown MycCaP-Bo cells were further selected by FACS sorting of mCherry^+^ cells after 300 ng/ml doxycycline treatment for 24 h. Quantitative PCR (qPCR) or immune-cellular staining were used to confirm the knockdown efficiency of shRNA and overexpression of *Inhba*.

#### Itga5-overexpressing MycCaP-Bo cells

gRNAs targeting the promoter of *Itga5* in murine cells were designed using the online tool at https://www.benchling.com/crispr. gRNAs were cloned into BbsI site of all-in-one vector (UniSAM vector, a kind gift from Prof. Lesley Forrester, University of Edinburgh, Edinburgh, UK) following the protocol described previously (Fidanza et al., 2017). The UniSAM vector includes elements of CAS9-VP64, MS2-p65-HSF1, and gRNA for gene expression activation, and mCherry for identification (Fidanza et al., 2017). Sequences for gRNA targeting murine *Itga5* (5′–3′) are: mItga5-1: 5′-TCC​TCT​CCG​CTT​CCC​CCT​CC-3′; mItga5-4: 5′-GGT​CTG​GCC​TGG​CTC​AGA​CT-3′.

MycCaP-Bo cells were transfected using Fugene HD (Roche) with 5 μg all-in-one dCAS9-SAM (UniSam) plasmid expressing single gRNA targeting the promoter of *Itga5* together with 5 μg pCMV-hyPBase (hyperactive transposase) for stable overexpression of *Itga5*. mCherry^+^ MycCaP-Bo cells were sorted out by FACS after 48 h of transfection. Flow cytometry was further used to examine the efficiency of *Itga5* overexpression.

### Mice

FVB/N was bought from Charles River. HiMyc mice were carried out in C. Sawyers’ lab at Memorial Sloan Kettering Cancer Center. B6.129S4-Ccr2tm1Ifc/J (*Ccr2*^−/−^) mice were bought from Jackson Laboratory and backcrossed with FVB/N mice to the 12th generation for experiments. CD11b-DTR mice were kindly provided from Richard Lang (The Children’s Hospital Research Foundation, Cincinnati, OH, USA). To generate CD11b-DTR bone marrow mosaic mouse, FVB/N mice (recipient mice) at the age of 3 wk received 9 Gy irradiation and rested for 5 h, followed by i.v. injection of 10^7^ bone marrow cells from CD11b-DTR mice (donor mice). These mice were allowed to recover for 3 wk before used for the subsequent experiments. CD169-DTR mice were kindly provided by Prof. Paul Frenette (Albert Einstein College of Medicine, New York, NY, USA). CD169-DTR mice originally in B6 background were crossed with athymic nude mice to allow tumor growth. All experiments involving mice were performed in accordance with United Kingdom Co-ordinating Committee on Cancer Research guidelines by approved protocol (P57A3693F). The study of mice was approved by the University of Edinburgh animal care and use committees.

### In vivo experiments

#### Bone metastasis formation and quantification

4- to 6-wk-old mice for all strains were used for bone metastasis assays. Mice received intracardiac injection of PC cells MycCaP-Bo (4 × 10^5^ cells/mouse) at day −7 to develop bone metastasis. Mice with bone metastasis detected by bioluminescence images (BLI) at day 0 were administered with different treatments according to diagram specified in each figure. The growth of bone metastasis was monitored by BLI twice a week using IMAGER OPTIMA system (Biospace). The quantification of bone metastasis growth was focusing on the signals from hind legs and normalized to BLI signal of day 0 (or day 14 as indicated) of same tumor to obtain the relative growth.

#### HiMyc primary tumor formation and quantification

HiMyc mice at age of ∼14-mo-old with palpable primary PC were used for subsequent treatments. The volume of tumor was measured using magnetic resonance imaging scanning once a week. The quantification of primary tumor growth was normalized to tumor volume of day 0 of same tumor to obtain the relative growth.

#### Drug treatments

Vehicle and enzalutamide (30 mg/kg body weight), SRC inhibitor eCF506 (20 mg/kg body weight) was given via daily oral gavage; L-Clod/PBS (1 mg/mouse, twice a week) were administered by i.v. injection; DT or control Glu^52^-DT (25 μg/kg body weight, every other day), anti-Ly-6G depleting Abs (200 μg/mouse, every other day), activin-A receptor inhibitor SB-505124 (5 mg/kg body weight, every other day) were delivered by i.p. injection. In some experiments, mice continuously received doxycycline diet (625 mg/kg). Enzalutamide was synthesized by chemical core at Memorial Sloan Kettering Cancer Center; eCF506 was kindly provided by A. Unciti-Broceta; L-Clod/PBS was from Liposoma; anti-Ly-6G Abs (clone #1A8) were from BioxCell; activin-A receptor inhibitor SB-505124 was from Selleckchem; and DT/Glu^52^-DT was from Sigma-Aldrich.

### Immuno-staining

#### Immunohistochemistry staining

Hind legs with bone metastasis were fixed by 4% paraformaldehyde (PFA; Sigma-Aldrich) overnight, decalcified by 14% EDTA for 7 d, followed by paraffin embedding. Primary tumors of HiMyc mice were fixed by 4% PFA overnight and embedded in paraffin. Sections with thickness of 5 μm were dewaxed by histoclear, antigen-retrieved in citrate buffer (pH 6.0), blocked with 5% normal goat-serum for 1 h at room temperature, and incubated with primary Abs against Iba1 (EPR16588, 1:500, #ab178846; Abcam), or cleaved caspase-3 (5A1E, 1:100, #9664S; Cell Signaling Technology) overnight at 4°C. On the next day, sections were incubated with secondary Abs and visualized with 4,4′-diaminobiphenyl using a VECTASTAIN Elite ABC-HRP Kit (Vector Laboratories). Nuclei were counterstained by hematotoxin. Images were taken under brightfield by Leica microscope.

#### Immunofluorescence (IF) staining

Staining with paraffin-embedded sections were performed the same as immunohistochemistry staining on the first day using primary Abs against Ki-67 (OTI5D7, 1:100, #ab156956; Abcam), or FN1 (1:500, NBP1-91258SS; Novus Biologicals). On the second day, sections were incubated AF555-conjugated secondary Abs (1:200, A-21434; Invitrogen) and counterstained with DAPI for nucleus. Images were taken using confocal Zeiss LSM 710 Microscope. Staining of FN1 was quantified as the area of positive signals divided by the filed area using ImageJ.

Inducible Ctrl/Fn1 knockdown MycCaP-Bo cells were seeded on poly-lysine coated coverslips, treated with doxycycline (300 ng/ml) for 4 d, and fixed by 4% PFA for 30 min. Cells were permeabilized using PBS containing 0.3% Triton X-100 and 1% BSA for 15 min, blocked in 10% normal goat serum for 1 h at room temperature, and incubated with primary Abs against FN1 (1:500, NBP1-91258SS; Novus Biologicals) at 4°C overnight, followed by AF488-conjugated secondary Abs and DAPI staining. Images were taken using a confocal Zeiss LSM 710 Microscope.

#### Whole mount staining

Hind legs with bone metastasis were fixed in 4% PFA for 30 min, followed by overnight soakage of 30% sucrose at 4°C. Samples were embedded in optimal cutting temperature compound and shaved by cryo-stat machine to expose bone marrow and bone metastasis lesion. Exposed samples were blocked with 5% BSA containing CD16/32 Ab (2.4G2, 1:200; BD Biosciences) for 1 h at room temperature, stained with primary Abs against Iba1 (EPR16588, 1:500, #ab178846; Abcam) and FITC-conjugated F4/80 (BM8, 1:50, #123108; BioLegend) at 4°C for 48 h, and incubated with AF555-conjugated secondary Abs against rabbit IgG (1:200, A-31572; Invitrogen) in the dark at room temperature for 2 h. Samples were counterstained with DAPI (1:1,000; Biomol) and then move to spinning disk for imaging.

### Cell culture and enzalutamide response

Murine PC cell line MycCaP-Bo cells or modified cells were maintained in DMEM (Life Technologies) supplemented with 10% FBS (Life Technologies) containing 1% penicillin-streptomycin (100 U/ml; Life Technologies).

#### Response to enzalutamide in vitro

Indicated cells (4 × 10^3^/well) were seeded into a 24-well plate with overnight coating of 1% BSA or 1 μg/ml fibronectin, followed by treatment of control (DMSO), enzalutamide (1 μM) for 4 d. Cells were incubated with 3-(4,5-dimethylthiazol-2-yl)-2,5 diphenyltetrazolium bromide (MTT reagent; 250 μg/ml; Sigma-Aldrich) at 37C for 1 h. The absorbance of DMSO-resolved solution at 540 nm (O.D. 540) was measured to determine the relative cell number. Response to enzalutamide was valued by dividing O.D. 540 from enzalutamide group over that from control group under the same well-coating condition.

#### IL-23 and MDSC-conditioned medium on enzalutamide resistance

IL-23 was purchased from Peprotech (200-23). Murine bone marrow MDSC–conditioned medium and enzalutamide-primed MDSC conditioned medium were harvested as previously described (Calcinotto et al., 2018). MycCaP-Bo cells (1 × 10^3^) were seeded into a 24-well plate and pre-treated with enzalutamide for 3 d, followed by normal culture (DMEM+10% FBS), IL-23 (100 ng/ml), or MDSC-conditioned medium, enzalutamide alone (1 μM), and IL-23 (100 ng/ml) or enzalutamide-primed MDSC-conditioned medium plus enzalutamide (1 μM). MTT assay was performed 4 d after the treatment to test the response of MycCaP-Bo cells to enzalutamide with indicated treatment.

### Flow cytometry analysis

#### Tumor and macrophage identification and sorting

Hind legs with bone metastasis were ground with digestion medium (DMEM supplemented with 100 μg/ml DNase I, 100 μg/ml Liberase TL, 100 μg/ml Liberase DL) and incubated at 37°C, 700 rpm for 30 min. Digested samples were single-cell filtered, lysed for red blood cells using red blood cell lysis buffer (420301; BioLegend), blocked with CD16/32 Abs (1:200; BD Bioscience), and stained for a combination of Abs against CD45 (30-F11, #103116; BioLegend), CD11b (M1/70, #101210; BioLegend), Ly-6C (HK1.4, #128033; BioLegend), Ly-6G (1A8, #127618; BioLegend), CCR2 (475301, FAB5538A; R&D System), F4/80 (BM8, #123146; BioLegend), CD169 (SER-4, #12-5755-82; Thermo Fisher Scientific), and CD106 (429(MVCAM.A), #105705; BioLegend). DAPI was used to exclude dead cells. Tumor cells were defined as CD45-iRFP^+^; macrophage was identified as CD45^+^CD11b^+^Ly-6C^−^Ly-6G^−^F4/80^+^SSC^low^. Cells were sorted on Aria II or Aria Fusion or analyzed on Fortessa (BD Bioscience).

In vivo samples with indicated treatment were stained with Ab against CD45 (30-F11, #103116; BioLegend), ITGA5 (HMα5-1, #103906; BioLegend).

### qPCR

Total RNA was extracted by (Sigma; #RTN350-1KT). cDNA was generated using QuantiTect Rev. Transcription Kit (#205313; Qiagen). qPCR was performed using RT^2^ SYBR Green ROX qPCR Mastermix Kit (#330523; Qiagen). qPCR assay was performed on ABI Quantstudio 5 machine and normalized to *Gapdh*.

Primers used for qPCR are listed as follows: *Gapdh*: 5′-F-TCA​CCA​CCA​TGG​AGA​AGG​C-3′, 5′-R-GCTAAGCAGTTGGTGGTGCA-3′; *Fn1*: 5′-F-CCC​AGC​TCA​CTG​ACC​TAA​GC-3′, 5′-R-TTCTCCTGCCGCAACTACTG-3′; *Inhba*: 5′-F-GGA​GAT​AGA​GGA​CGA​CAT​TGG​C-3′, 5′-R-CTGGTTCTGTTAGCCTTGGGG-3′; *Itga5*: 5′-F-CTT​CTC​CGT​GGA​GTT​TTA​CCG-3′, 5′-R-GCTGTCAAATTGAATGGTGGTG-3′; *Acvr1b*: 5′-F-GTG​GGG​ACC​AAA​CGA​TAC​ATG-3′, 5′-R-CTGGTCACATACAACCTTTCGC-3′; *Acvr2a*: 5′-F-GGG​ACG​CAT​TTC​TGA​GGA​TAG-3′, 5′-R-GCCATTCCTGCATGTTTCTGC-3′;

### Western blot

Protein from MycCaP-Bo–derived bone metastasis with indicated treatments or cultured in vitro with various conditions was extracted by grinding tissues using radioimmunoprecipitation assay lysis buffer (Thermo Fisher Scientific) supplemented with protease and phosphatase inhibitors (Roche). The concentration of protein was measured by BCA assay using Kit (Thermo Fisher Scientific). 50 μg of total protein was loaded into each well for SDS/PAGE and transferred to polyvinylidene difluoride membranes (Millipore). After blocking in Odyssey Blocking Buffer TBS (927-50000; LI-COR Biosciences), membranes were probed with primary Abs against pSRC (D49G4, #6943; Cell Signaling Technology) or SRC (36D10, #2109; Cell Signaling Technology) overnight at 4°C. Membrane was washed in Odyssey Blocking Buffer TBS three times and incubated with 680RD conjugated donkey anti-rabbit IgG Ab (926-68073; LI-COR Biosciences) for 1 h at room temperature or incubated with HRP-linked anti-rabbit IgG Ab (#7074; Cell Signaling Technology) for 1 h at room temperature. Membrane was detected using Infra-red Imager LI-COR Odyssey Fc chemi system.

### Bulk RNA-seq and differential expression analysis

Tumor cells, monocytes, and macrophages from bone metastasis with different treatments were sorted by flow cytometry according to the identification of CD45-iRFP^+^ (tumor cells), CD45^+^CD11b^+^Ly-6C^hi^CCR2^+^ (monocytes), and CD45^+^CD11b^+^Ly-6C^−^Ly-6G^−^F4/80^+^SSC^low^ (macrophages), respectively. Tumor cells, monocytes, and macrophages from three mice were pooled together for total RNA extraction using RNAeasy micro plus kit (catalog #74034; Qiagen) following the manufacturer’s instructions. Quality control was performed using Bioanalyser Picokit (Agilent Technologies). RNAs from tumor cells were sequenced on BGISEQ-500 Platform as paired-end reads at the Beijing Genomics Institute (BGI), China. Differentially expressed genes were determined using NOISeq (Tarazona et al., 2015; Tarazona et al., 2012). Genes with fold change > ±1.5 and probability >0.8 were defined as differentially regulated. Novel genes (probes starting with “BGI_novel”) were removed from subsequent analysis.

### scRNA-seq

#### Sample preparation

Hind legs from healthy mouse or bone metastasis mouse were harvested and processed as for flow cytometry analysis. After red blood cell lysis, the cells were pelleted by centrifuged and resuspended in 0.1% BSA PBS. Samples with more 90% live cells were concentrated to 700–1,000 cells/μl and further loaded onto Chromium Single-Cell Instrument (10X Genomics) to generate single-cell gel bead-in-emulsions targeting a recovery of 5,000–6,000 cells according to Chromium Single Cell 3′ Reagent Kits instruction (10X Genomics).

#### scRNA-seq library preparation and sequencing

Library construction was performed using the Chromium Single Cell 3′ Reagent Kit version 2 and 3 (10X Genomics). BGISEQ-500 sequencer (BGI) was applied to sequence the scRNA-seq library with a strategy of 26 bp of read-1 (10X barcode and randomer), 100 bp of read-2, and 8 bp of barcodes.

#### scRNA-seq data processing

CellRanger Software Suite (version 2.0 and 3.0, 10X Genomics) was used to generate a raw gene expression matrix for each scRNA-seq sample with all default parameters. Scrublet (Wolock et al., 2019) was used to infer and remove cell doublets in each sample individually. Then the gene expression matrices of all samples were combined in R (version 4.0.4; https://www.r-project.org) and were processed with Seurat R package (version 4.0.2; Hao et al., 2021). Quality filtering was performed to remove cells with <201 or >9,000 expressed genes or >25% unique molecular identifiers derived from the mitochondrial genome. In the remaining cells, gene expression matrices were log normalized to total cellular read-counts and mitochondrial read-counts by linear regression implemented using the “ScaleData” function of the Seurat package.

#### Cell type identification

To reduce dimensionality, principal component analysis was used to summarize the resulting variably expressed genes. The batch effects were removed by the Harmony package (version 1.0; Korsunsky et al., 2019) based on the top 15 principal components. Then the top 15 principal components were further summarized using UMAP (Becht et al., 2018) to present data in a two-dimensional panel. Clusters were identified by an shared nearest neighbor modularity optimization-based clustering algorithm (Waltman and Van Eck, 2013). The clusters were annotated based on the differentially expressed genes in each cluster and the well-known cellular markers from the literature.

#### Differential gene expression analysis

To identify differentially expressed genes for subtypes, the functions “FindAllMarkers” (multiple condition comparisons) from the Seurat package were used with default parameters. Significant differentially expressed genes (markers) were selected as those with adjusted P values <0.05, average twofold-change larger than 0.6 and percentage of cells with gene expression detected in at least 0.1 of cells in either one of the two comparison groups.

#### Pseudotime analysis

The Monocle3 R package (version 1.0.0; Qiu et al., 2017; Cao et al., 2019) was used to estimate a pseudotemporal path of five subsets of macrophages. A monocle function “DifferentialGeneTest” was used to detect genes with differential expression between clusters, and the top 2,000 with a q-value <0.01 were selected to construct the single cell trajectories.

### Bioinformatics analysis

Bioinformatics analysis was performed with R and Bioconductor (Gentleman et al., 2004). Data were visualized using ggplot2, GGally, and VennDiagram libraries. Gene annotation was performed using database of The Database for Annotation, Visualization and Integrated Discovery (DAVID; Huang et al., 2009) and clusterProfiler (Yu et al., 2012).

A dataset (GSE32269; Cai et al., 2013) with hormone-dependent primary PC and castration-resistant metastatic PC and datasets (GSE101607 [Ylitalo et al., 2017], GSE29650 [Hornberg et al., 2011], GSE74685 [Haider et al., 2016], and GSE66187 [Zhang et al., 2015]) with castration-resistant PC bone metastasis samples and multiple organ metastases were analyzed. Two datasets with gene expression data from primary PCa samples and clinical data on lethality (GSE16560; Sboner et al., 2010) and biochemical recurrence (GSE21032; Taylor et al., 2010) were used in GSEA.

The cell type enrichment analysis tool xCell (Aran et al., 2017) was used to calculate stromal cell enrichment scores. Macrophage content was estimated using ImSig deconvolution tool (Nirmal et al., 2018). Pearson correlation analysis was used to analyze the correlation between ssGSEA scores, immune cell abundance, and gene expression.

For survival analysis, a SU2C dataset downloaded from cBioPortal (https://www.cbioportal.org/) with overall survival and time on treatment (enzalutamide/abiraterone) information was used (Abida et al., 2019). Samples were stratified using mean and median gene expression and type of treatment where relevant. Cox proportional hazards model was used to calculate significance and hazard ratio values.

Downstream pathway activities were calculated using ssGSEA (Barbie et al., 2009) scores of pathway target gene lists using the Gene Set Variation Analysis package (Hanzelmann et al., 2013). Gene symbols were converted to human or mouse homologs where required using HomoloGene. To calculate ECM score, KEGG homo sapiens ECM–receptor interaction pathway (04512) with a total of 84 genes was used. To measure Src pathway activity, differentially upregulated genes (log_2_FC > 2 and P < 0.05) in v-Src overexpressed primary prostate epithelial cell established from the ventral prostates of FVB mice were determined using publicly available gene expression data (GSE37428) and RankProduct analysis (Hong et al., 2006).

#### Statistical analysis

All experimental data shown (excluding bioinformatics analysis) were generated using GraphPad Prism software and displayed as mean ± SEM or median ± quartiles. Statistical comparisons were performed using two-tailed unpaired Student’s *t* test. *, P < 0.05 was considered significant. Two-tailed Student’s *t* test and ANOVA with post-hoc Tukey honest significant difference for multiple comparison was used to calculate statistical significance for bioinformatics analysis.

### Online supplemental material

Fig. S1 shows that PC bone metastasis–associated neutrophils, basophils, mast cells, and endothelial cells are not inversely correlated with patient survival. Fig. S2 shows that neutrophils contribute minimally to anti-androgen resistance. Fig. S3 shows macrophage sub-populations in bone-metastatic PC. Fig. S4 shows that macrophage-mediated upregulation of FN1, but not LAMB2, in tumor cells is highly enriched in bone metastasis. Fig. S5 shows *INHBA*, but not *CNTF*, correlated with macrophage content.

## Supplementary Material

SourceData F7 contains original files for Fig. 7.

## Data Availability

RNA-seq data have been deposited in the National Center for Biotechnology Information’s Gene Expression Omnibus under accession no. GSE156427. scRNA-seq data are available at the China National GeneBank DataBase under accession no. CNP0003856.
