# Supplementary figures and images for: Macrophages promote anti-androgen resistance in prostate cancer bone disease
Source: J Exp Med. 2023 Feb 7;220(4):e20221007. doi: 10.1084/jem.20221007 (PMC9948761; doi:10.1084/jem.20221007)

Figure 7G

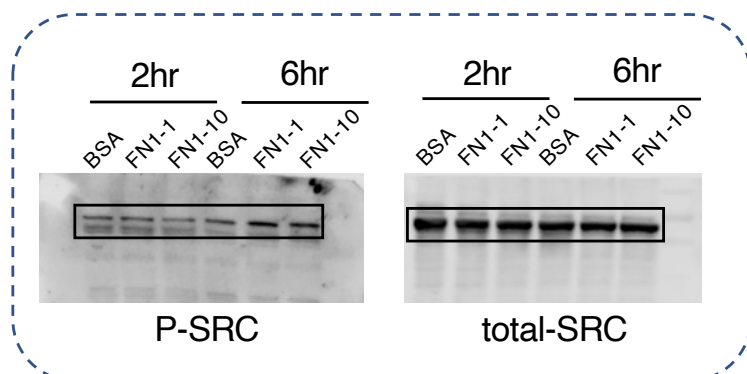

Figure 7H

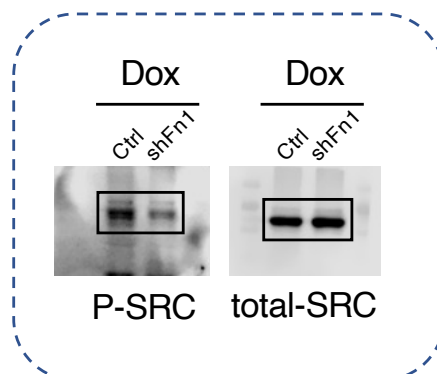

Figure 7I

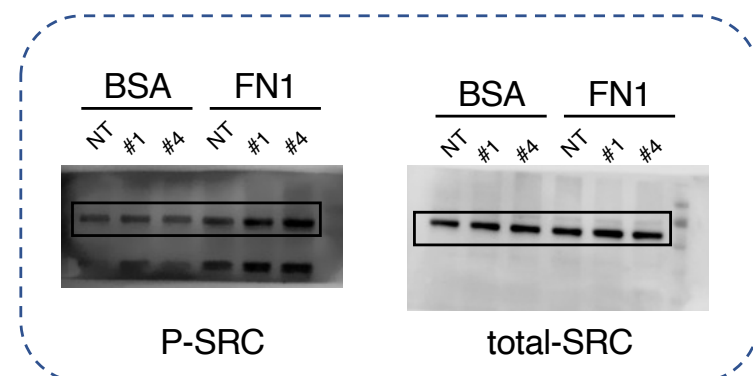

Figure 7J

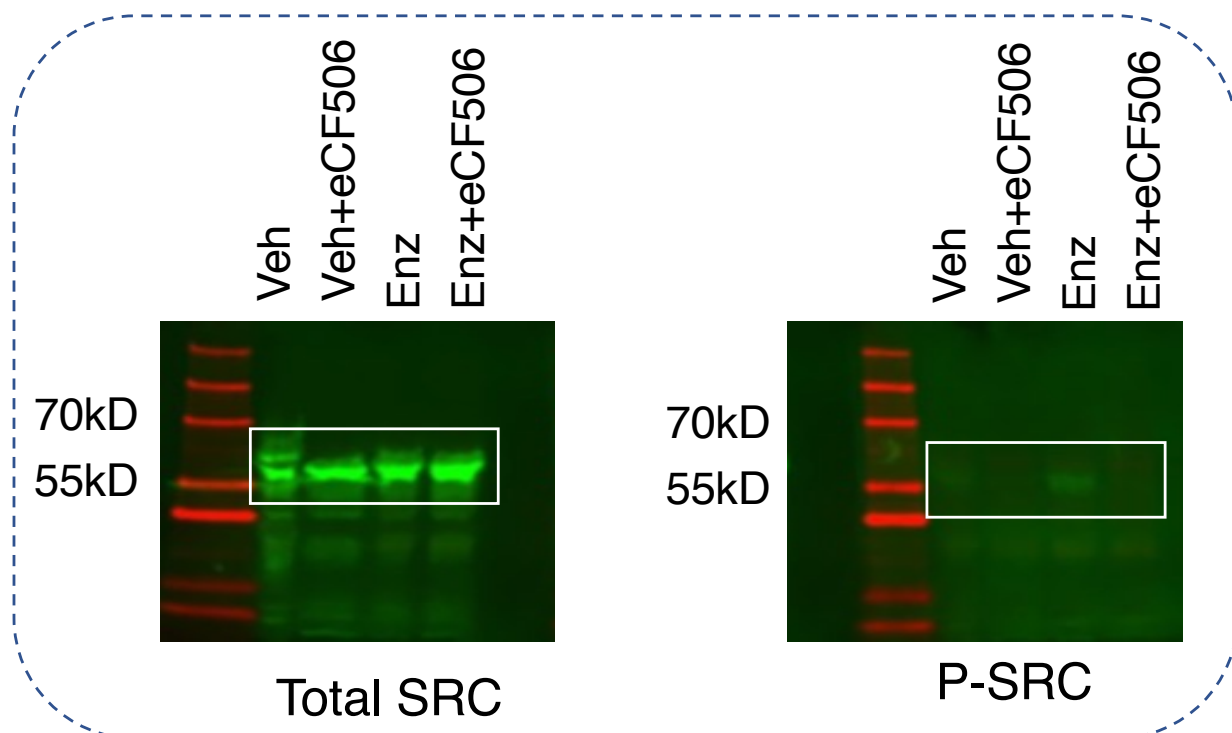

Supplement: SourceData F7 — contains original files for Fig. 7. [file JEM_20221007_SourceDataF7.pdf]
